# Discovery of an Unconventional Centromere in Budding Yeast Redefines Evolution of Point Centromeres

**DOI:** 10.1016/j.cub.2015.06.023

**Published:** 2015-08-03

**Authors:** Norihiko Kobayashi, Yutaka Suzuki, Lori W. Schoenfeld, Carolin A. Müller, Conrad Nieduszynski, Kenneth H. Wolfe, Tomoyuki U. Tanaka

**Affiliations:** 1 Centre for Gene Regulation and Expression, College of Life Sciences, University of Dundee, Dundee DD1 5EH, UK; 2 Department of Computational Biology, School of Frontier Medicine, University of Tokyo, Chiba 277-8562, Japan; 3 Whitehead Institute for Biomedical Research, Cambridge, MA 02142, USA; 4 Howard Hughes Medical Institute and Department of Biology, Massachusetts Institute of Technology, Cambridge, MA 02139, USA; 5 Sir William Dunn School of Pathology, University of Oxford, Oxford OX1 3RE, UK; 6 UCD Conway Institute, School of Medicine and Medical Science, University College Dublin, Dublin 4, Ireland

## Abstract

Centromeres are the chromosomal regions promoting kinetochore assembly for chromosome segregation. In many eukaryotes, the centromere consists of up to mega base pairs of DNA. On such “regional centromeres,” kinetochore assembly is mainly defined by epigenetic regulation [1]. By contrast, a clade of budding yeasts (*Saccharomycetaceae*) has a “point centromere” of 120–200 base pairs of DNA, on which kinetochore assembly is defined by the consensus DNA sequence [2, 3]. During evolution, budding yeasts acquired point centromeres, which replaced ancestral, regional centromeres [4]. All known point centromeres among different yeast species share common consensus DNA elements (*CDE*s) [5, 6], implying that they evolved only once and stayed essentially unchanged throughout evolution. Here, we identify a yeast centromere that challenges this view: that of the budding yeast *Naumovozyma castellii* is the first unconventional point centromere with unique *CDE*s. The *N. castellii* centromere *CDE*s are essential for centromere function but have different DNA sequences from *CDE*s in other point centromeres. Gene order analyses around *N. castellii* centromeres indicate their unique, and separate, evolutionary origin. Nevertheless, they are still bound by the ortholog of the CBF3 complex, which recognizes *CDE*s in other point centromeres. The new type of point centromere originated prior to the divergence between *N. castellii* and its close relative *Naumovozyma dairenensis* and disseminated to all *N. castellii* chromosomes through extensive genome rearrangement. Thus, contrary to the conventional view, point centromeres can undergo rapid evolutionary changes. These findings give new insights into the evolution of point centromeres.

## Highlights

• A new type of point centromere has been identified in budding yeast *N. castellii*
• Its DNA sequence and evolutionary origin are different from other point centromeres
• *N. castellii* centromeres are bound by CBF3 that recognizes other point centromeres
• Contrary to the conventional view, point centromeres can change rapidly in evolution

## Graphical Abstract

**Figure:**
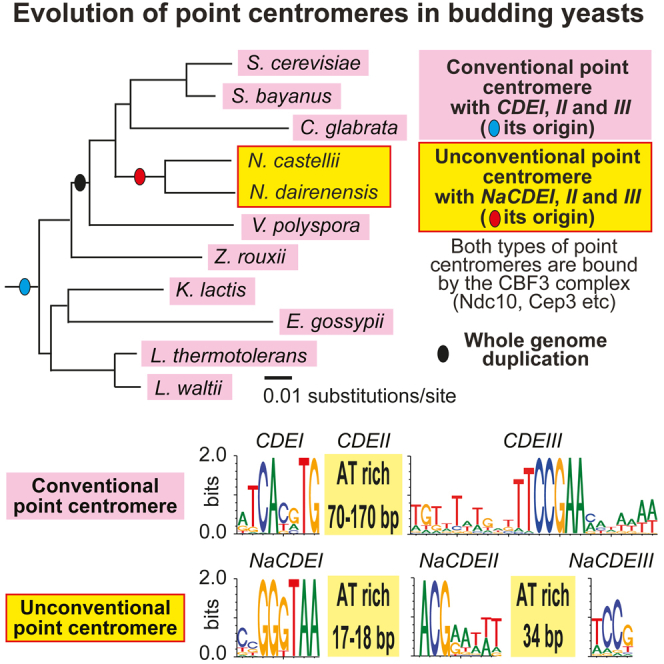

## Teaser

All known point centromeres share common DNA sequences and a single evolutionary origin. Kobayashi et al. have identified a new type of point centromere in budding yeast *N. castellii*. Its DNA sequence and evolutionary origin are different from other point centromeres. Discovery of the new centromere redefines the evolution of point centromeres.

## Results and Discussion

**Figure 1:**
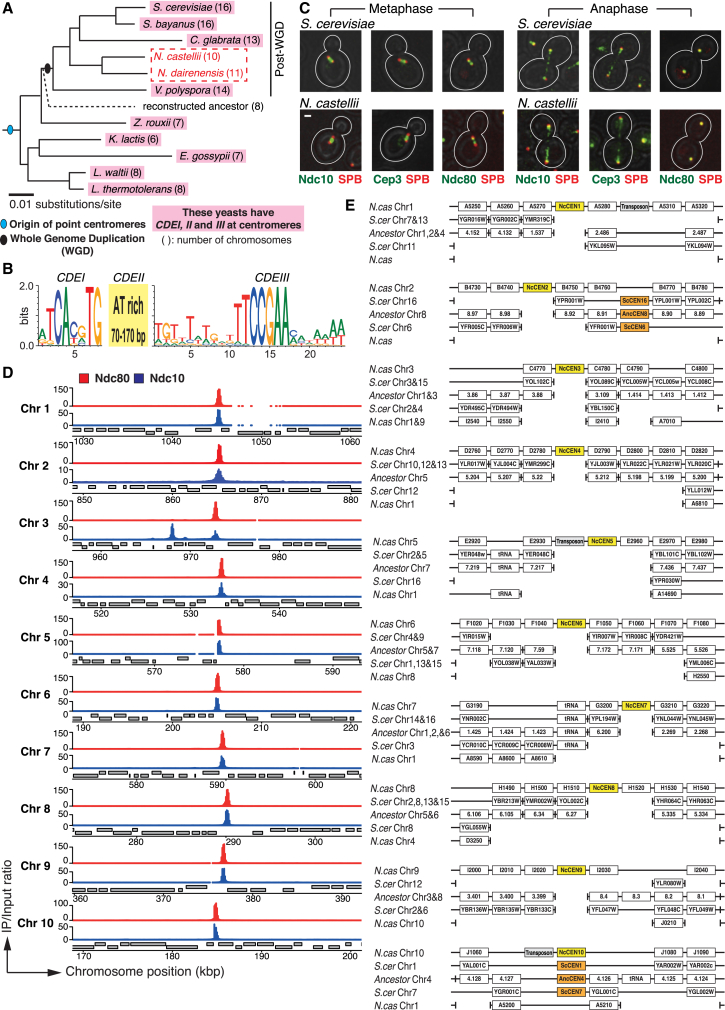
Kinetochore Proteins Show Single-Peak Localization on Each Chromosome of *N. castellii* (A) A clade of budding yeast species carries point centromeres with common consensus DNA sequences *CDEI*, *II*, and *III* (pink), except for *N. castellii* and *N. dairenensis* (red rectangle). Phylogenetic tree of budding yeast species is taken from [6, 7] with modification. Horizontal length in the tree is proportional to the change in genomic DNA sequences. The WGD (black oval) is estimated to have occurred approximately 100 million years ago [8], and the *N. castellii/N. dairenensis* divergence is about half this age. (B) Nucleotide sequence of *CDEI*, *II*, and *III* [5, 6]. Logos of nucleotides graphically represent their frequency at individual positions. *CDEII* is 70–170 bp DNA sequence with >79% AT content. (C) Localization of Ndc10, Cep3, and Ndc80 in representative *S. cerevisiae* and *N. castellii* cells (in metaphase and anaphase). Ndc10, Cep3, and Ndc80 were tagged with three tandem copies of GFP (3× GFP) in *N. castellii* (T11587, T11584, and T11586, respectively) and with 1× GFP in *S. cerevisiae* (T11520, T11522, and T11521, respectively). Spindle pole body (SPB) components, Spc42 and Spc110, were tagged with 4× mCherry and 1× mCherry in *N. castellii* and *S. cerevisiae,* respectively. Cell shapes are outlined in white. The scale bar represents 1 μm. Metaphase and anaphase are defined by the distance between two SPBs (<2.5 μm and >3 μm, respectively). Note that, during anaphase, CBF3 also localizes on the spindle as well as at kinetochores [9]. (D) Ndc80 (red) shows single-peak localization on each chromosome (Chr) of *N. castellii*. Ndc10 (blue) also shows peaks at the same regions. *NDC80-6xHA* (T9328) and *NDC10-6xHA* (T9326) cells were processed for ChIP-seq. Gray bars represent open reading frames of genes; top on Watson strand, bottom on Crick strand. Chromosome positions are shown in length (kilo base pairs [kbp]) from the left telomere. In addition to the positions of Ndc80 peaks, Ndc10 showed two extra peaks (Figure S1A); one of them is at 968 kbp on chromosome 3, as shown here. (E) Gene order in *S. cerevisiae* and the reconstructed ancestor, aligned around *N. castellii CEN*s (yellow box). Gene orders were analyzed using YGOB [10]. Vertical tick bars represent gaps; i.e., genes to the right and left are not neighbors on a chromosome. Two chromosome series of *N. castellii* and *S. cerevisiae* are aligned with one series of the ancestor because *N. castellii* and *S. cerevisiae* are post-WGD yeasts (see A). Orange boxes represent centromeres in the ancestor (*AncCEN*) and *S. cerevisiae* (*ScCEN*). See also Figure S1.

All known point centromeres have a common DNA sequence, known as *CDEI*, *II*, and *III* (Figures 1A and 1B) [5, 6]. Among different budding yeast species, the order of orthologous genes is generally conserved around the centromeres (i.e., in synteny) [11]. These indicate that the point centromeres themselves are orthologous. However, there are peculiar exceptions to this view: *Naumovozyma castellii* (*N. castellii*) (previously called *Saccharomyces castellii*) and its close relative *N. dairenensis* belong to the clade of budding yeasts expected to have point centromeres [3, 7], but no *CDEI*,*II*,*III*-like sequences are found at the loci expected from synteny (Figure 1A; see Figure S1D) [6, 12]. They may use a novel type of point centromere or have re-acquired regional centromeres during their evolution. Here, we aim to identify the centromere in *N. castellii*.

### Kinetochore Components Show Single-Peak Localization on Each Chromosome of *N. castellii* by Chromatin Immunoprecipitation

In *Saccharomyces cerevisiae* (*S. cerevisiae*) and other budding yeasts, their point centromeres are recognized by the CBF3 complex, which binds the *CDEIII* DNA consensus [5, 13]. The CBF3, consisting of Ndc10, Cep3, Ctf13, and Skp1 proteins, is exclusively found in budding yeasts with point centromeres. In *S. cerevisiae*, the CBF3 and other kinetochore components show a bi-lobed pattern on the metaphase spindle and segregate following movement of the spindle poles during anaphase [14] (Figure 1C, top). Despite an apparent lack of *CDEI*,*II*,*III*-containing centromeres, the *N. castellii* genome encodes orthologs of the CBF3 components [10, 15]. In *N. castellii*, Ndc10 and Cep3 proteins showed the kinetochore-like localization pattern, similar to *S. cerevisiae* (Figure 1C, bottom). The same localization pattern was found for Ndc80 (Figure 1C, bottom), an outer kinetochore component [5]. Thus, Ndc10, Cep3, and Ndc80 might indeed be *N. castellii* kinetochore components.

To identify *N. castellii* centromeres, we added epitope tags to Ndc10, Cep3, and Ndc80 at their original loci, carried out chromatin immunoprecipitation followed by high-throughput DNA sequencing (ChIP-seq), and analyzed in reference to the annotated *N. castellii* genome sequence [15, 16]. We also carried out ChIP-seq for Cse4, a centromere-specific histone H3 variant. Crucially, Ndc80 ChIP-seq gave a distinct single peak at an intergenic region on each of ten chromosomes (Figures 1D and S1A). Cse4, Ndc10, and Cep3 gave peaks at the same ten intergenic regions as Ndc80 and gave one, two, and six additional peaks, respectively (Figures 1D and S1A–S1C). The chromosomal regions, where Ndc80 showed accumulation (together with Ndc10, Cep3, and Cse4), may serve as the centromeres in *N. castellii*. On this assumption, we tentatively named them *N. castellii CEN1–10*, or *NcCEN1–10* for short, on chromosomes 1–10, respectively.

### Most *N. castellii CEN*s Are Not at Conserved Syntenic Locations on Chromosomes, Compared with Locations of Other Point Centromeres

We compared the order of orthologous genes between *S. cerevisiae*, *N. castellii*, and an ancestor. This ancestor is evolutionarily positioned prior to the whole-genome duplication (WGD), which occurred during the evolution of budding yeasts [3] (Figure 1A), and its genome was constructed using bioinformatics [17]. Between *S. cerevisiae* and the ancestor, the gene order across the centromeres is conserved, including the centromeres themselves [11, 17] (Figure S1D). Thus, chromosomal positions of the centromeres did not change during evolution from the ancestor to *S. cerevisiae*. The gene order across the majority of the ancestral centromeric regions (excluding the centromeres themselves) is also conserved without rearrangement on *N. castellii* chromosomes (Figure S1D). However, *N. castellii CEN* locations are not syntenic to the *CEN*s in the ancestor (Figures S1D and S1E). This suggests that centromeres “disappeared” from these ancestral centromeric loci during the *N. castellii* evolution. In fact, ancestral *CEN4* and *N. castellii CEN10* make the only centromere pair whose surrounding orthologs show complete synteny (Figures S1D and S1E). Conversely, *N. castellii CEN1–9* are not located in regions of conserved gene order when compared to *S. cerevisiae* or the ancestor (Figure 1E). More specifically, the synteny along *N. castellii* chromosomes (relative to those of *S. cerevisiae* and the ancestor) is disrupted at the positions of *NcCEN*s. It is therefore likely that *N. castellii CEN1–9* have been positioned, at least partly, by genome rearrangement during evolution, rather than by de novo centromere formation between the existing genes.

### Candidate Centromere Regions Show Dynamic Behaviors Expected for Functional Centromeres in *N. castellii* Cells

**Figure 2:**
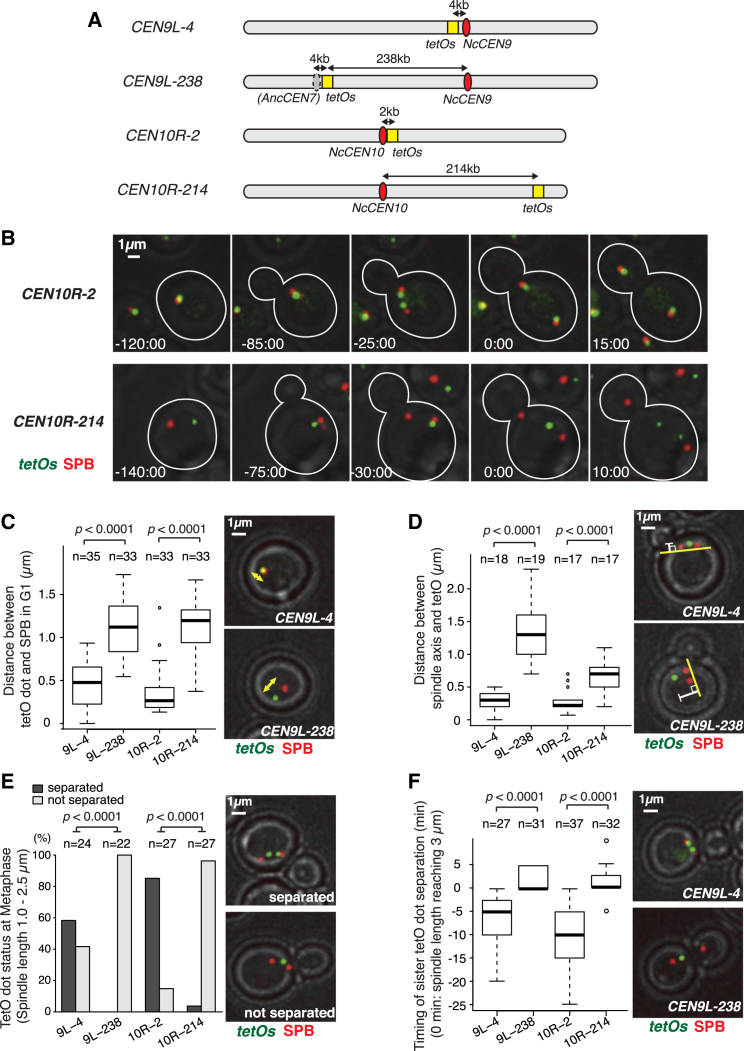
*N. castellii* Candidate Centromeres Show Dynamic Localizations during the Cell Cycle, which Are Consistent with Those of Functional Centromeres (A) Diagram showing positions of the insertion of *tetO* arrays (112 tandem repeats; yellow box) on chromosomes 9 and 10. *NcCEN9* and *NcCEN10* are shown as red ovals. The corresponding position of the ancestor *CEN7* (*AncCEN7*), based on synteny, is shown as a gray oval. (B) Live-cell images of *tetO* arrays shown in (A). Images of *SPC42-4xmCherry TetR-GFP* cells with *tetOx112* at *CEN10R-2* (T11466) or at *CEN10R-214* (T11467) were acquired every 5 min. Spc42 is an SPB component. Time (min: s) is shown relative to anaphase onset (when the distance between SPBs exceeded 3 μm). The scale bar represents 1 μm. (C–F) Analyses of *tetO*s localization in cells. Live-cell images of *SPC42-4xmCherry TetR-GFP* cells with *tetOx112* at *CEN9L-4* (T11501), *CEN9L-238* (T11500), *CEN10R-2* (T11466), or *CEN10R-214* (T11467) were acquired every 5 min. (C) Distance between the SPB and the *tetO* dot in G1 phase is shown (in cells with one SPB but no bud). In box plots, a thick line represents a median; a box shows the range of the first to third quartile (interquartile range: IQR); the upper and lower whiskers show the maximum and minimum values, respectively, which do not exceed 3/2 IQR beyond the box; and open circles show outliers. (D) Distance between the *tetO* dot and the spindle axis in metaphase is shown (cells with two SPBs less than 2 μm apart and with a single *tetO* dot). Box plots are as in (C). (E) Frequency of separation and non-separation of the *tetO* dot in metaphase is shown (cells with two SPBs 1.0–2.5 μm apart). (F) Timing of separation of the *tetO* dot, relative to anaphase onset, is shown (as defined in B). Representative cells show separation of sister *tetO* dots (at *CEN9L-4*) before anaphase onset (top) and no separation of them (at *CEN9-L-238*) after anaphase onset (bottom). Box plots are as in (C).

We addressed whether *N. castellii CEN*s show expected localizations of functional centromeres in cells. If *N. castellii CEN*s promote kinetochore assembly, spindle microtubules should attach and apply forces on these chromosome regions. In *S. cerevisiae*, such forces cause separation of sister chromatids up to 10 kb from centromeres on the metaphase spindle [18–21]. To investigate this in *N. castellii*, we inserted *tet* operators at 2–4 kb from *NcCEN9* (*CEN9L-4*) and *NcCEN10* (*CEN10R-2*; Figure 2A). As controls, we also inserted the *tet* operators on chromosome arms (*CEN9L-238* and *CEN10R-214*). *CEN9L-238* is also positioned at 4 kb right of the locus corresponding to the ancestor *CEN7*, based on synteny. The *tet* operators were bound by TetR-GFP fusion proteins [22] and visualized as small GFP dots (Figure 2B). In G1 phase, GFP dots at *CEN9L-4* and *CEN10R-2* localized in the vicinity of the spindle pole body (SPB), whereas those at *CEN9L-238* and *CEN10R-214* were at a larger distance from the SPB (Figure 2C). In metaphase, GFP dots at *CEN9L-4* and *CEN10R-2* located near the axis defined by two SPBs (Figure 2D) and often showed two signals, indicative of sister chromatid separation (Figure 2E). In contrast, the GFP dots at *CEN9L-238* or *CEN10R-214* did not separate until early anaphase (Figures 2E and 2F), whereas the GFP dots at *CEN9L-4* and *CEN10R-2* moved immediately after SPB segregation during anaphase (Figure 2B, top). These behaviors of GFP dots at *CEN9L-4* and *CEN10R-2* are similar to those at the *S. cerevisiae* centromeres [18–21] and consistent with *NcCEN*s indeed being functional centromeres in *N. castellii*.

### *N. castellii CEN*s Include Unique Consensus DNA Elements, which Are Crucial for Minichromosome Propagation

**Figure 3:**
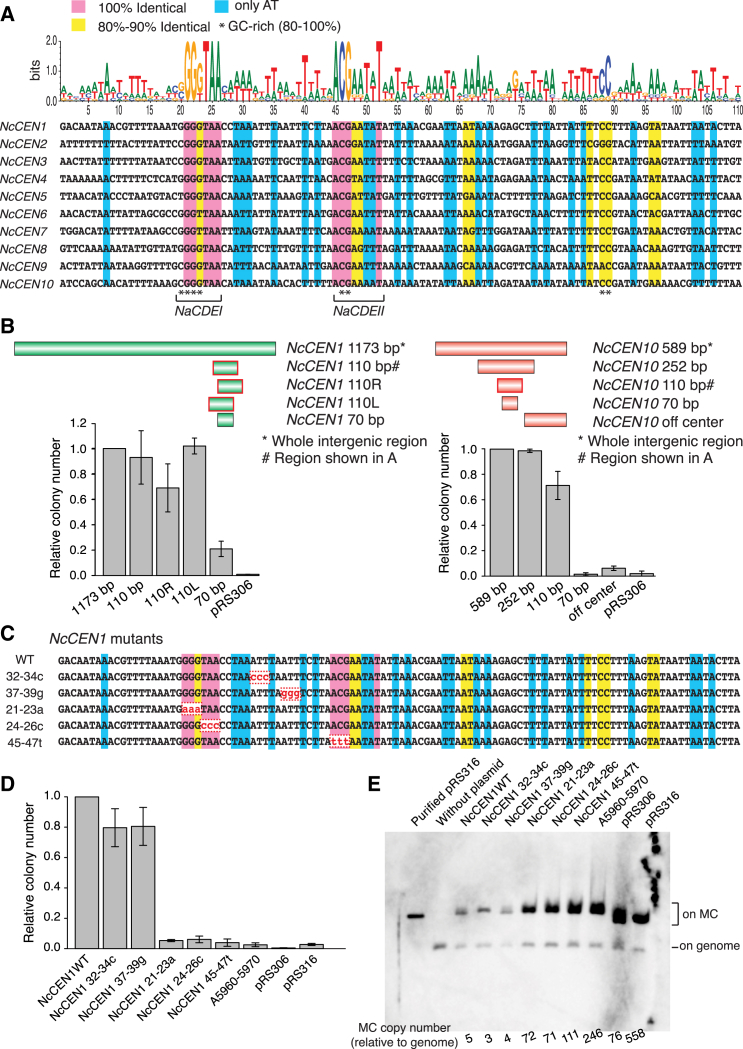
*N. castellii CEN*s Have Unconventional Consensus DNA Elements, which Are Important for Minichromosome Propagation (A) Consensus DNA sequence found at *N. castellii CEN*s. Logos of nucleotides (top) graphically represent their frequency at individual positions. Nucleotide positions, highlighted in pink and yellow, represent those identical in 100% and 80%–90% *NcCEN*s, respectively. Blue shows positions only with A and T, whereas an asterisk shows GC-rich (80%–100%) positions. Two highly conserved elements were named *NaCDEI* (position 20–26) and *NaCDEII* (position 45–52), which are SGGKTAA (S: G or C; K: G or T) and ACGDDWWT (D: not C; W: A or T), respectively. (B) Consensus DNA sequence of 110 bp shows full centromere activity for minichromosome (MC) propagation. The DNA fragments shown in the diagram (top) were inserted into the *pRS306* plasmid, which carries *S. cerevisiae URA3* [23] that works as a selection marker in *N. castellii* cells [24]. These DNA constructs were introduced into *N. castellii* haploid cells with *ura3-1* (T11421) for colony formation assay. *NcCEN1* 1,173 bp and *NcCEN10* 589 bp cover the whole intergenic region containing a Ndc80-enriched region (Figure 1D). *NcCEN1* 110 bp and *NcCEN10* 110 bp are shown in (A), whereas *NcCEN1* 70 bp and *NcCEN10* 70 bp correspond to positions 20–89 in (A). *NcCEN1* 110 bp DNA sequence was shifted to right and left by 20 bp along the chromosome, making *NcCEN1* 110R and 110L, respectively. Colony numbers are normalized to that with *NcCEN1* 1,173 bp and *NcCEN10* 589 bp. Error bars represent SEM (n = 3). (C) Mutations introduced to *NcCEN1* DNA sequence (110 bp shown in A). WT, wild-type. (D) Colony formation assay using *NcCEN1* mutants shown in (C). The number of *N. castellii* colonies with a MC carrying each mutant (on 1,173 bp wild-type *NcCEN1*; see B) was normalized to that with wild-type *NcCEN1* (1,173 bp; see B). The *A5690*–*A5970* intergenic DNA fragment (1,071 bp) from a chromosome 1 arm was also integrated into a MC and used as a negative control. Error bars represent SEM (n = 3). (E) Copy number (per cell) of each MC was evaluated using Southern blots. Genomic and MC DNA was digested by *Not* I, separated by electrophoresis, blotted, and probed with the ampicillin resistance gene (the host strain has one copy of it at *hoΔ* allele on the genome). The number at the bottom shows a ratio of each MC to the genome (ratio of intensity of hybridized bands). See also Figure S2.

We compared DNA sequences at *NcCEN1–10*, which include Ndc80-enriched regions in ChIP-seq (Figure 1D). Approximately in the middle of the enriched regions, we identified *NcCEN* consensus DNA sequences (Figures 3A and S2A). In particular, two short DNA elements showed very high similarity in all ten *NcCEN*s at positions 20–26 and 45–52 in Figure 3A, which we name *NaCDEI* and *NaCDEII* (*Naumovozyma* consensus DNA element), respectively. In addition, other regions showed similarity or common AT- or GC-rich areas among *NcCEN*s. The overall consensus within the 70-bp sequence at positions 20–89 in Figure 3A is unique to *NcCEN*s and found at no other regions in the *N. castellii* genome. Remarkably, the consensus found at *N. castellii CEN*s is very different from the consensus of other point centromeres (*CDEI*, *II*, and *III*; Figure 1B).

We evaluated the activity of candidate centromeres on minichromosomes in *N. castellii* cells. If minichromosomes were able to undergo both DNA replication and mitotic segregation, they are stably propagated during cell proliferation. DNA replication origins have not yet been identified in *N. castellii*, and we investigated the propagation of *pRS306* and *pRS316* plasmids, on which an *S. cerevisiae* centromere and replication origin are absent and present, respectively [23]. *pRS306* and *pRS316* were maintained at high copy number in *N. castellii* cells (>70 per cell; see Figure 3E). Yeast cells accumulate minichromosomes with high copy number if their replication occurs normally but segregation is inefficient [25]. We reasoned that, in *N. castellii* cells, an *S. cerevisiae* centromere does not promote minichromosome segregation efficiently, but its replication is supported, even without an *S. cerevisiae* replication origin (Figures S2B–S2D). Notably, addition of *NcCEN1* to *pRS306* caused a marked reduction in its copy number to 3–5 per cell (see Figure 3E; *NcCEN1* WT) and the formation of many more yeast colonies (Figure 3B; *NcCEN1* 1,173 bp). Addition of *NcCEN10* showed similar effects (Figure 3B; *NcCEN10* 589 bp), whereas addition of a control, chromosome arm DNA fragment (A5960–5970) had no such effect (see Figures 3D and 3E). Thus, *N. castellii CEN*s are able to facilitate minichromosome propagation and yeast colony formation, presumably by promoting mitotic segregation.

To determine the minimum DNA sequence carrying a centromere activity, we examined the ability of several DNA fragments within *NcCEN1* and *NcCEN10* to support minichromosome propagation (Figure 3B). The 70-bp sequence at position 20–89 in Figure 3A was essential for centromere activity, and additional 20- to 40-bp sequences around it facilitated the activity. The 20- to 40-bp sequences are not particularly similar among the *NcCEN*s (Figure 3A), but their AT richness may contribute to their centromere activity (Table S1) as does the AT-rich *CDEII* in other budding yeasts [13]. Subsequently, we addressed whether the consensus DNA elements *NaCDEI* and *NaCDEII* are important for centromere activity. Three base-pair mutations within the *NaCDEI* (*21–23a* and *24–26c*) and *NaCDEII* (*45–47t*) of *NcCEN1* (Figure 3C) showed substantial decreases in yeast colony formation (Figure 3D) and high copy numbers of minichromosomes (>70 per cell), indicative of inefficient segregation (Figure 3E). Three base-pair control mutations between *NaCDEI* and *NaCDEII* (*32–34c* and *37–39 g*) showed similar numbers of yeast colonies to wild-type *NcCEN1* (Figure 3D) and maintained low copy numbers of minichromosomes (3–5 per cell; Figure 3E). Thus, *NaCDEI* and *NaCDEII* are important for the centromere activity.

Using these assays, we next evaluated requirement of RNAi for centromere activity in *N. castellii*. This pathway is present in some budding yeasts, including *N. castellii* [26], and required for centromere activity in fission yeast [27]. The centromere activity for minichromosome propagation was still normal without the RNAi pathway in *N. castellii* (Figures S2E and S2F).

### Consensus DNA Elements in *N. castellii CEN*s Promote CBF3 Binding and Facilitate Centromere Activity on Authentic Chromosomes

**Figure 4:**
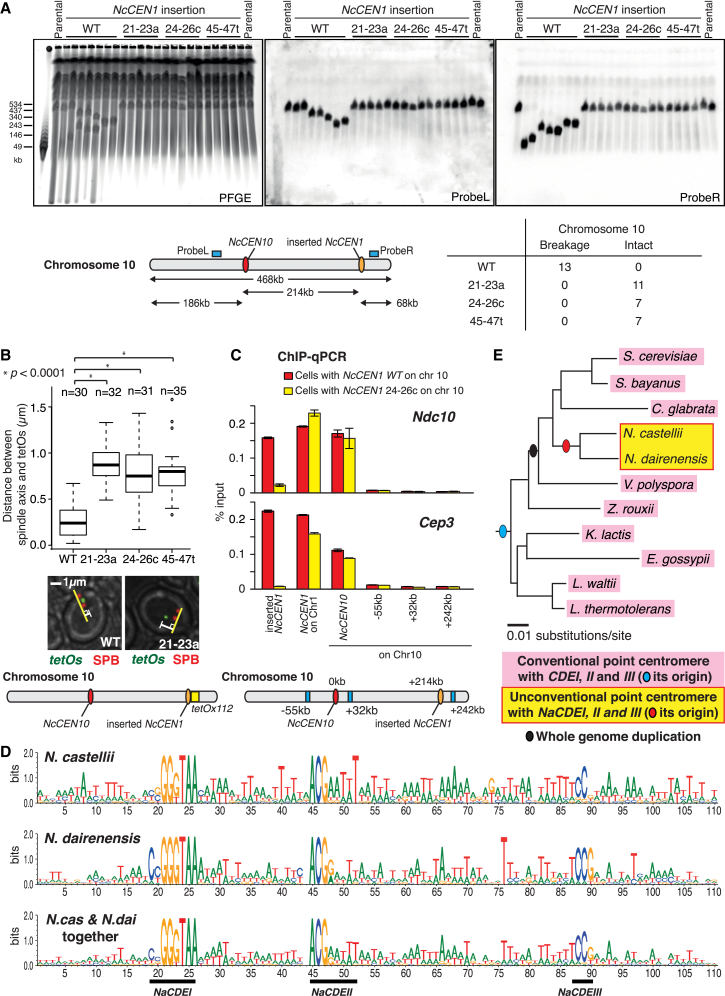
Consensus DNA Elements in *N. castellii CEN*s Are Important for the Centromere Function in the Context of Authentic Chromosomes (A) *NcCEN1* wild-type (WT; 1,173 bp; Figure 3B) and its mutants (1,173 bp with 21–23a, 24–26c, and 45–47t; Figure 3C) were inserted at 214 kb right of *NcCEN10* on chromosome 10 (orange oval in diagram) in haploid *N. castellii* cells (T11605). Karyotypes of individual clones were analyzed (representative examples are shown here) by pulsed field gel electrophoresis (PFGE), followed by Southern blots with ProbeL and ProbeR; see positions of the probes in diagram. Table shows the number of clones that did, or did not, show breakage of chromosome 10 (shortening detected by ProbeL and/or ProbeR; Figure S3A). (B) *NcCEN1* wild-type (WT; T11632), 21–23a (Figure 3C; T11842), 24–26c (T11843), and 45–47t (T11844), each marked with *tetOx112*, were inserted at 214 kb right of *NcCEN10* on chromosome 10 in *SPC42-4xmCherry TetR-GFP* cells. Cells were imaged, and the distance between the spindle axis and inserted *NcCEN1* was analyzed as in Figure 2D. Box plots are as in Figure 2C. (C) *NDC10-3xFLAG* cells with inserted *NcCEN1* wild-type (WT; T11845) and 24–26c (T11846), as well as *CEP3-3xFLAG* cells with inserted *NcCEN1* wild-type (WT; T11847) and 24–26c (T11848), were processed for ChIP-qPCR. Diagram shows chromosome loci for quantification by PCR (blue box). Primers for qPCR were designed to distinguish the original *NcCEN1* on chromosome 1 and *NcCEN1* inserted on chromosome 10. Error bars represent SEM (n = 3). (D) *N. dairenensis* candidate centromeres (Figure S4B) have consensus DNA elements, *NaCDEI*, *II*, and *III*, which are also found at *N. castellii CEN*s (Figure 3A). Logos of nucleotides graphically represent their frequency at individual positions, in aligned *N. castellii* centromeres (top), *N. dairenensis* candidate centromeres (middle), and both together (bottom). (E) Conclusion of this study: budding yeasts, highlighted in pink, have point centromeres with *CDEI*, *II*, and *III*, which are conserved across several species. In contrast, *N. castellii* and *N. dairenensis*, highlighted in yellow, carry unconventional point centromeres with *NaCDEI*, *II*, and *III*, which have very different DNA sequences from conventional *CDEI*, *II*, and *III*. Blue and red ovals show the origins of the two types of point centromere in the yeast phylogenetic tree. See also Figures S3 and S4.

We next addressed whether the centromere DNA elements identified above are also crucial for centromere activity on authentic chromosomes. When an additional active centromere is inserted into a yeast chromosome, it causes chromosome breakage between the original and the newly inserted centromere [28, 29]. We employed this procedure to assess the centromere activity in *N. castellii*. We inserted wild-type and mutated *NcCEN1s* on the chromosome 10 arm (Figure 4A, diagram) and analyzed breakage of this chromosome using pulsed field gel electrophoresis (PFGE), followed by Southern blotting (Figure 4A). After insertion of a wild-type *NcCEN1*, all of 13 randomly chosen clones showed breakage of chromosome 10 (Figures 4A and S3A). By contrast, no such breakage was observed after insertion of *NcCEN1* carrying mutations at *NaCDEI* and *NaCDEII* (*21–23a*, *24–26c*, and *45–47t*). Next, we visualized the intracellular localization of wild-type and mutated *NcCEN1*s, inserted on chromosome 10. Wild-type *NcCEN1* on chromosome 10 was near SPBs during telophase to G1 (Figure S3B, left) and on the metaphase spindle with frequent sister separation (Figures 4B and S3B, right). However, mutated *NcCEN1* (*21–23a*, *24–26c*, and *45–47t*) did not show such behavior. Thus, *NaCDEI* and *NaCDEII* are crucial for centromere activity on authentic chromosomes.

As shown earlier, CBF3 components Ndc10 and Cep3 bind *NcCEN*s (Figures 1D and S1C). To address whether this binding requires *NaCDE*s, we used ChIP followed by qPCR (ChIP-qPCR; Figure 4C). Both Ndc10 and Cep3 showed enrichment at the original *NcCEN1* and wild-type *NcCEN1* inserted on chromosome 10, but not at the mutated *NcCEN1* on chromosome 10 (Figure 4C). Thus, the consensus DNA elements within *NcCEN* are required for CBF3 binding. Furthermore, we found both Ndc10 and Cep3 are essential genes in *N. castellii* (Figure S3C), as is expected if they have central roles in recognizing *NcCEN*s.

How can the CBF3 complex, which recognizes standard *CDE I*,*II*,*III*-type *CEN*s, also bind *NcCEN*s despite the different DNA sequences? We investigated the evolutionary conservation of Ndc10 and Cep3, the CBF3 components recognizing consensus DNA elements in budding yeasts [5, 13]. A putative DNA-binding domain of Cep3 is conserved between *N. castellii* and other budding yeasts [30]. By contrast, the core DNA-binding domain of Ndc10 showed a more-rapid change during evolution of *N. castellii*, compared with other budding yeasts with standard *CEN*s (Figures S3D–S3F). Such a rapid change may have happened to adapt to the new type of point centromere.

### *N. dairenensis*, a Close Relative of *N. castellii*, Has *N. castellii*-like Consensus DNA Elements at Its Candidate Centromere Regions

We next aimed to identify candidate centromeres in *N. dairenensis*, a close relative to *N. castellii* (Figure 1A). Based on the annotated *N. dairenensis* genome [16], we found that the orders of orthologous genes around most *N. castellii CEN*s are conserved on *N. dairenensis* chromosomes (Figure S4A). Crucially, at the corresponding intergenic regions, we identified *CDE*s that are very similar to *NaCDEI*, *II* found at *N. castellii CEN*s (Figures 4D and S4B). It is likely that these regions serve as *N. dairenensis* centromeres, but we could not test this prediction because of a lack of molecular genetics methods in *N. dairenensis.* This analysis revealed a third sequence element with evolutionary conservation (*NaCDEIII*; Figure 4D), but mutagenesis showed that it is not essential for centromere function in *N. castellii* (Figure S4B legend). *N. dairenensis* Ndc10 showed evolutionary changes, similarly to *N. castellii* Ndc10 (Figures S3D–S3F). In conclusion, *N. castellii CEN*s and *N. dairenensis* candidate centromeres have very similar consensus DNA elements (Figure 4D). The new type of centromere *CDE*s (*NaCDEI*, *II*, and *III*) originated prior to the branching point of *N. castellii* and *N. dairenensis* in evolution (Figure 4E).

### Conclusions

We have identified centromeres in the budding yeast *N. castellii*. We conclude that they make point centromeres, because (1) consensus DNA elements are found among all ten centromeres, (2) these DNA elements are important for the centromere activity, and (3) a short DNA fragment (110 bp) containing the consensus DNA elements is sufficient for centromere function. Crucially, the consensus centromere DNA elements are very different from those in other known point centromeres, highlighting the *N. castellii* centromere as the first unconventional, i.e., non-*CDE I*,*II*,*III*-type, point centromere (Figure 4E). The gene order analyses give the following insights: first, most *N. castellii* centromeres are not located in intergenic regions orthologous to those containing standard *CDEI*,*II*,*III*-type point centromeres in other species (Figure S1D), although these two are often in close proximity (Figure S4C). This indicates that these *N. castellii* centromeres did not descend from standard point centromeres at their individual chromosome regions. Second, at most *N. castellii* centromeres, synteny is disrupted when compared with the ancestral budding yeast genome (Figure 1E). This can be explained if *N. castellii* centromeres were propagated to all chromosomes during evolution through extensive genome rearrangement. The origin of the *N. castellii* centromere is still elusive, but it may have been propagated and superseded the conventional point centromeres.

## Accession Numbers

DNA sequences have been deposited under the following accession codes: ChIP-seq of Ndc80-6xHA, DDBJ: DRA002836; Ndc10-6xHA, DDBJ: DRA003502; Cep3-3xFLAG, DDBJ: DRA002836; Cse4-6xHA, DDBJ: DRA003067; *N. castellii CEN1-10*, DDBJ: LC029901–LC029910; and *N. dairenensis CEN1-11*, DDBJ: BR001264–BR001274.

## Supplemental Information

Document S1. Supplemental Experimental Procedures, Figures S1–S4, and Tables S1–S4

Document S2. Article plus Supplemental Information
