# Supplementary material for: Discovery of an Unconventional Centromere in Budding Yeast Redefines Evolution of Point Centromeres
Source: Curr Biol. 2015 Aug 3;25(15):2026–33. doi: 10.1016/j.cub.2015.06.023 (PMC4533239; doi:10.1016/j.cub.2015.06.023)
Supplement: Document S1. Supplemental Experimental Procedures, Figures S1–S4, and Tables S1–S4 [file mmc1.pdf]

Current Biology

Supplemental Information

Discovery of an Unconventional Centromere in Budding Yeast Redefines Evolution of Point Centromeres

Norihiko Kobayashi, Yutaka Suzuki, Lori W. Schoenfeld, Carolin A. Müller, Conrad Nieduszynski, Kenneth H. Wolfe, and Tomoyuki U. Tanaka

Fig S1A

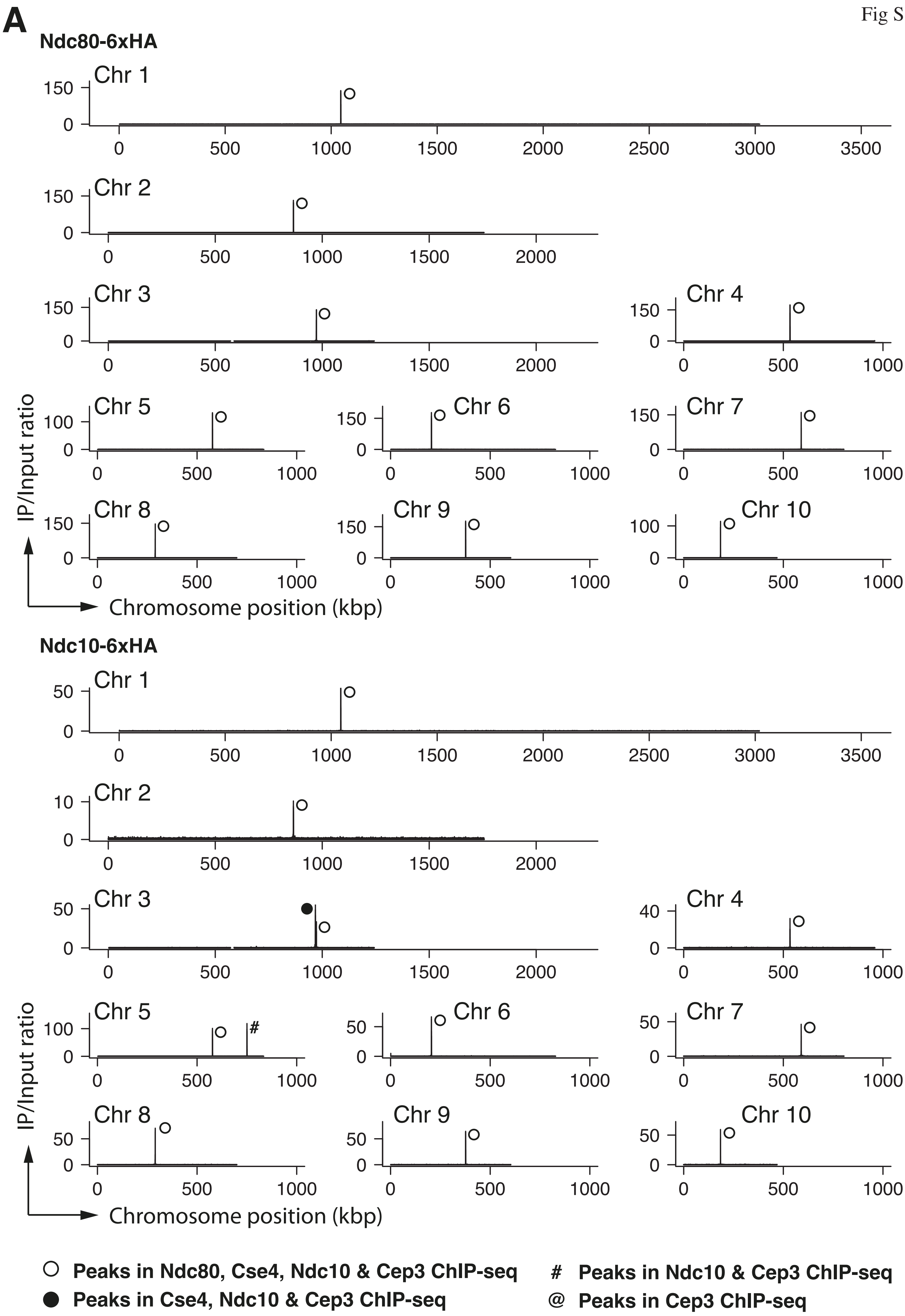
A
Ndc80-6xHA
Chr 1
Chr 2
Chr 3
Chr 4
Chr 5
Chr 6
Chr 7
Chr 8
Chr 9
Chr 10
IP/Input ratio
Chromosome position (kbp)
Ndc10-6xHA
Chr 1
Chr 2
Chr 3
Chr 4
Chr 5
Chr 6
Chr 7
Chr 8
Chr 9
Chr 10
IP/Input ratio
Chromosome position (kbp)
Peaks in Ndc80, Cse4, Ndc10 & Cep3 ChIP-seq
Peaks in Cse4, Ndc10 & Cep3 ChIP-seq
Peaks in Ndc10 & Cep3 ChIP-seq
@ Peaks in Cep3 ChIP-seq

Fig S1B

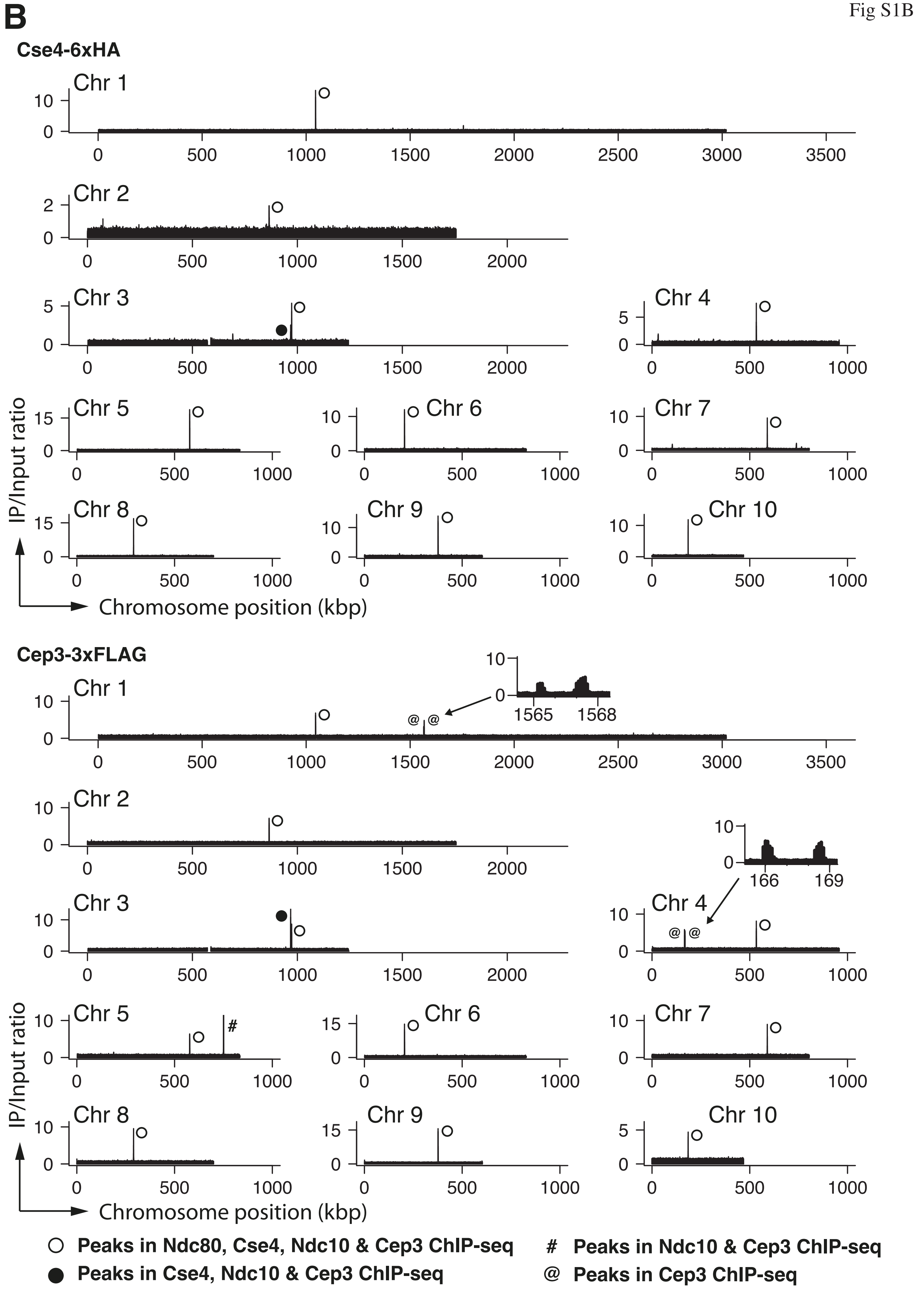

B
Cse4-6xHA
Chr 1
Chr 2
Chr 3
Chr 4
Chr 5
Chr 6
Chr 7
Chr 8
Chr 9
Chr 10
IP/Input ratio
Chromosome position (kbp)
Cep3-3xFLAG
Chr 1
1565
1568
Chr 2
166
169
Chr 3
Chr 4
Chr 5
Chr 6
Chr 7
Chr 8
Chr 9
Chr 10
IP/Input ratio
Chromosome position (kbp)
○ Peaks in Ndc80, Cse4, Ndc10 & Cep3 ChIP-seq
● Peaks in Cse4, Ndc10 & Cep3 ChIP-seq
Peaks in Ndc10 & Cep3 ChIP-seq
@ Peaks in Cep3 ChIP-seq

Fig S1C-E

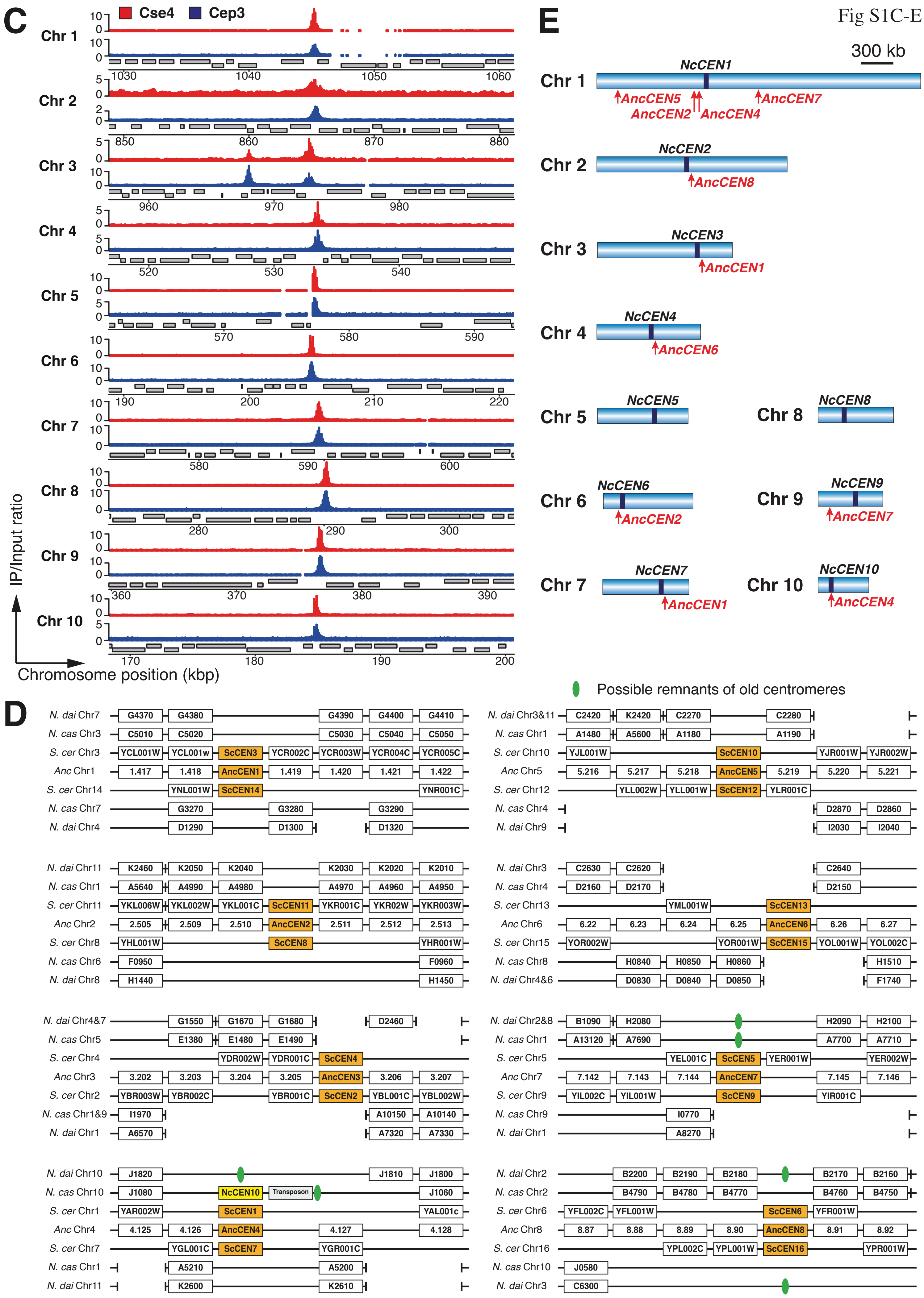
C
Cse4
Cep3
Chr 1
Chr 2
Chr 3
Chr 4
Chr 5
Chr 6
Chr 7
Chr 8
Chr 9
Chr 10
1030 1040 1050 1060
850 860 870 880
960 970 980
520 530 540
570 580 590
190 200 210 220
580 590 600
280 290 300
360 370 380 390
170 180 190 200
IP/Input ratio
Chromosome position (kbp)
E
300 kb
Chr 1 NcCEN1 AncCEN5 AncCEN2 AncCEN4 AncCEN7
Chr 2 NcCEN2 AncCEN8
Chr 3 NcCEN3 AncCEN1
Chr 4 NcCEN4 AncCEN6
Chr 5 NcCEN5
Chr 8 NcCEN8
Chr 6 NcCEN6 AncCEN2
Chr 9 NcCEN9 AncCEN7
Chr 7 NcCEN7 AncCEN1
Chr 10 NcCEN10 AncCEN4
Possible remnants of old centromeres
D
N. dai Chr7 G4370 G4380 G4390 G4400 G4410
N. cas Chr3 C5010 C5020 C5030 C5040 C5050
S. cer Chr3 YCL001W YCL001w ScCEN3 YCR002C YCR003W YCR004C YCR005C
Anc Chr1 1.417 1.418 AncCEN1 1.419 1.420 1.421 1.422
S. cer Chr14 YNL001W ScCEN14 YNR001C
N. cas Chr7 G3270 G3280 G3290
N. dai Chr4 D1290 D1300 D1320
N. dai Chr3&11 C2420 K2420 C2270 C2280
N. cas Chr1 A1480 A5600 A1180 A1190
S. cer Chr10 YJL001W ScCEN10 YJR001W YJR002W
Anc Chr5 5.216 5.217 5.218 AncCEN5 5.219 5.220 5.221
S. cer Chr12 YLL002W YLL001W ScCEN12 YLR001C
N. cas Chr4 D2870 D2860
N. dai Chr9 I2030 I2040
N. dai Chr11 K2460 K2050 K2040 K2030 K2020 K2010
N. cas Chr1 A5640 A4990 A4980 A4970 A4960 A4950
S. cer Chr11 YKL006W YKL002W YKL001C ScCEN11 YKR001C YKR02W YKR003W
Anc Chr2 2.505 2.509 2.510 AncCEN2 2.511 2.512 2.513
S. cer Chr8 YHL001W ScCEN8 YHR001W
N. cas Chr6 F0950 F0960
N. dai Chr8 H1440 H1450
N. dai Chr3 C2630 C2620 C2640
N. cas Chr4 D2160 D2170 D2150
S. cer Chr13 YML001W ScCEN13
Anc Chr6 6.22 6.23 6.24 6.25 AncCEN6 6.26 6.27
S. cer Chr15 YOR002W YOR001W ScCEN15 YOL001W YOL002C
N. cas Chr8 H0840 H0850 H0860 H1510
N. dai Chr4&6 D0830 D0840 D0850 F1740
N. dai Chr4&7 G1550 G1670 G1680 D2460
N. cas Chr5 E1380 E1480 E1490
S. cer Chr4 YDR002W YDR001C ScCEN4
Anc Chr3 3.202 3.203 3.204 3.205 AncCEN3 3.206 3.207
S. cer Chr2 YBR003W YBR002C YBR001C ScCEN2 YBL001C YBL002W
N. cas Chr1&9 I1970 A10150 A10140
N. dai Chr1 A6570 A7320 A7330
N. dai Chr2&8 B1090 H2080 H2090 H2100
N. cas Chr1 A13120 A7690 A7700 A7710
S. cer Chr5 YEL001C ScCEN5 YER001W YER002W
Anc Chr7 7.142 7.143 7.144 AncCEN7 7.145 7.146
S. cer Chr9 YIL002C YIL001W ScCEN9 YIR001C
N. cas Chr9 I0770
N. dai Chr1 A8270
N. dai Chr10 J1820 J1810 J1800
N. cas Chr10 J1080 NcCEN10 Transposon J1060
S. cer Chr1 YAR002W ScCEN1 YAL001c
Anc Chr4 4.125 4.126 AncCEN4 4.127 4.128
S. cer Chr7 YGL001C ScCEN7 YGR001C
N. cas Chr1 A5210 A5200
N. dai Chr11 K2600 K2610
N. dai Chr2 B2200 B2190 B2180 B2170 B2160
N. cas Chr2 B4790 B4780 B4770 B4760 B4750
S. cer Chr6 YFL002C YFL001W ScCEN6 YFR001W
Anc Chr8 8.87 8.88 8.89 8.90 AncCEN8 8.91 8.92
S. cer Chr16 YPL002C YPL001W ScCEN16 YPR001W
N. cas Chr10 J0580
N. dai Chr3 C6300

Figure S1. Supplemental Figures associated with Figure 1.

(A) Genome-wide ChIP-seq data of Ndc80 and Ndc10. *NDC80-6xHA* (T9328) and *NDC10-6xHA* (T9326) cells were processed for ChIP-seq and results are shown genome wide. Chromosome positions are shown as length (kilo base pair; kbp) from the left telomere. Ndc80 ChIP gave a distinct single peak (open circles) at an intergenic region on each of all 10 chromosomes (Figure 1D). Ndc10 ChIP gave 10 peaks at the same regions as Ndc80 (open circles; Figure 1D) and, in addition, two extra peaks; one of the two extra peaks locates between C4740 and C4750 on chromosome 3 (closed circle; Figure 1D), the other is between E3810 and E3820 on chromosome 5 (marked with #).

(B) Genome-wide ChIP-seq data of Cse4 and Cep3. *CSE4-6xHA* (T9377) and *CEP3-3xFLAG* (T11450) cells were processed for ChIP-seq and results are shown genome wide. Chromosome positions are shown as length (kilo base pair; kbp) from the left telomere. Cse4 ChIP gave 10 peaks at the same regions as Ndc80 (open circles; Figure S1C) and, in addition, one extra peak that is in common with Ndc10 ChIP (closed circle, between C4740 and C4750 on chromosome 3; Figure S1C). Cep3 ChIP gave 10 peaks at the same regions as Ndc80 (open circles; Figure S1C) and, in addition, six extra peaks; one is in common with Ndc10 and Cse4 ChIP (closed circle, between C4740 and C4750 on chromosome 3; Figure S1C), one is common with Ndc10 but not with Cse4 (marked with #, between E3810 and E3820 on chromosome 5), and four are unique in Cep3 ChIP (two twin peaks, marked with @ and also shown in a magnified scale; between A7860 and A7870 and between A7870 and A7880 on chromosome 1, between D0880 and D0890 and between D0890 and D0900 on chromosome 4). The intergenic region between C4740 and C4750 on chromosome 3, where a peak is found commonly in Ndc10, Cse4 and Cep3 ChIP (closed circle; Figures 1D, S1C), contains DNA sequence GGGTAA + 18 nucleotides + AAG, which is very similar (but not identical) to centromere DNA consensus *NaCDEI* and *NaCDEII* (see Figure 3A). Other extra peaks in Ndc10 and Cep3 ChIP (marked with # and @) do not contain DNA sequence that is obviously similar to *NaCDE*s, and the reason for Ndc10 and Cep3 accumulation is currently unclear.

(C) ChIP-seq data of Cse4 and Cep3 around the regions with Ndc80 peaks. ChIP-seq results of Cse4 (red) and Cep3 (blue) are shown in the same chromosome regions as shown in Figure 1D. Gray bars represent open reading frames of genes; top on Watson strand, bottom on Crick strand. Chromosome positions are shown as length (kilo base pair; kbp) from the left telomere. In addition to ten intergenic regions with Ndc80 peaks (on each of ten chromosomes; Figure 1D), Cse4 showed an extra peak at 968 kbp on chromosome 3 (where Cep3 also showed a peak), as shown in this figure. Data are absent at some chromosome regions where DNA sequence is repetitive (e.g. around 1050 kbp on chromosome 1).

(D) Gene order in *S. cerevisiae*, *N. castellii* and *N. dairenensis*, aligned around the centromeres of the reconstructed ancestral yeast. Gene orders were analyzed using YGOB [S1-3]. Orange boxes show centromeres in the reconstituted ancestor (*AncCEN*) [S2] and *S. cerevisiae* (*ScCEN*). Yellow boxes show *N. castellii CENs* (*NcCEN*). Vertical tick bars represent gaps, i.e. genes at their right and left are not neighbors on a chromosome. Two chromosome series of *S. cerevisiae* (*S. cer*), *N. castellii* (*N. cas*) and *N. dairenensis* (*N. dai*) are aligned with one series of the ancestor (*Anc*), because *S. cerevisiae, N. castellii* and *N. dairenensis* are post-WGD yeasts (Figure 1A).

This figure, together with Figure 1E, suggests that *N. castellii*'s *CEN* locations are not syntenic to the *CEN*s in the ancestor. Indeed, the gene order across the majority of

the ancestral centromeric regions (i.e. from their left to right along a chromosome) — regions around *AncCEN1, 2, 4, 5, 7* and *8* — is also conserved without rearrangement on *N. castellii* chromosomes (excluding the *CEN*s themselves), but *N. castellii*'s *CEN* locations are not syntenic to the *CEN*s in the ancestor. For example, the inferred centromere of ancestral chromosome 2 (*AncCEN2*) lies between genes Anc_2.510 and Anc_2.511, which correspond to genes *A4980* and *A4970* on *N. castellii* chromosome 1. However, the ChIP-seq data show that the centromere of *N. castellii* chromosome 1 is between genes *A5270* and *A5280*, about 53 kb away from the ancestral site (Figure 1E).

Note that we found two and four possible remnants of old centromeres on the *N. castellii* and *N. daire*nensis genome, respectively (green ovals), in the following intergenic regions that correspond to the location of the ancestral *CEN*s (based on synteny):

A7690–A7700 (*N. castellii*); TTCCGAA

J1060–transposon (*N. castellii*); ATCCGAA

J1810–J1820 (*N. dairenensis*); CAGCTG (214 bp; 70%AT) TTCCGAA

H2080–H2090 (*N. dairenensis*); CATATG (75 bp; 69%AT) TACCGAA

B2170–B2180 (*N. dairenensis*); AACCGAA

C6300–C6310 (*N. dairenensis*); CACGTG (59 bp; 68%AT) TACCGAA

All of them contain core *CDEIII*-like sequences (WWCCGAA [W: A or T]), and three of them are accompanied by the *CDEI*-like sequence (CAXXTG) (see Figure 1B). The two *CDEIII*-like sequence in *N. castellii* were not bound by the Ndc80 Ndc10, Cse4 or Cep3 in ChIP-seq.

(E) Positions of candidate *N. castellii* centromeres on chromosomes. Diagram of *N. castellii* chromosomes, showing presumed locations of centromeres. *AncCEN1* etc indicate corresponding centromere positions in a reconstructed ancestor (see text), in cases where gene order is conserved at ancestral centromere sites (see **A**). Because of the WGD, each centromere in the ancestral genome should in principle map onto two sites in the *N. castellii* genome, but due to rearrangements some of them cannot be mapped.

Note that, while we were preparing this manuscript, Koszul's group predicted the positions of *N. castellii* centromeres genome-wide [S4]. They used a chromosome conformation capture technique, based on the assumption that centromeres should locate closely to a spindle pole. In their study, *NcCEN3* and *NcCEN9* were mapped to the same intergenic regions as those in our study (Figures 1D, S1C). In contrast, other centromeres were positioned 0.6–3.0 kb away from ours, in the next intergenic region or on a neighboring open reading frame etc. This difference is probably due to a higher resolution of our ChIP-seq method (Figure 1D, S1C).

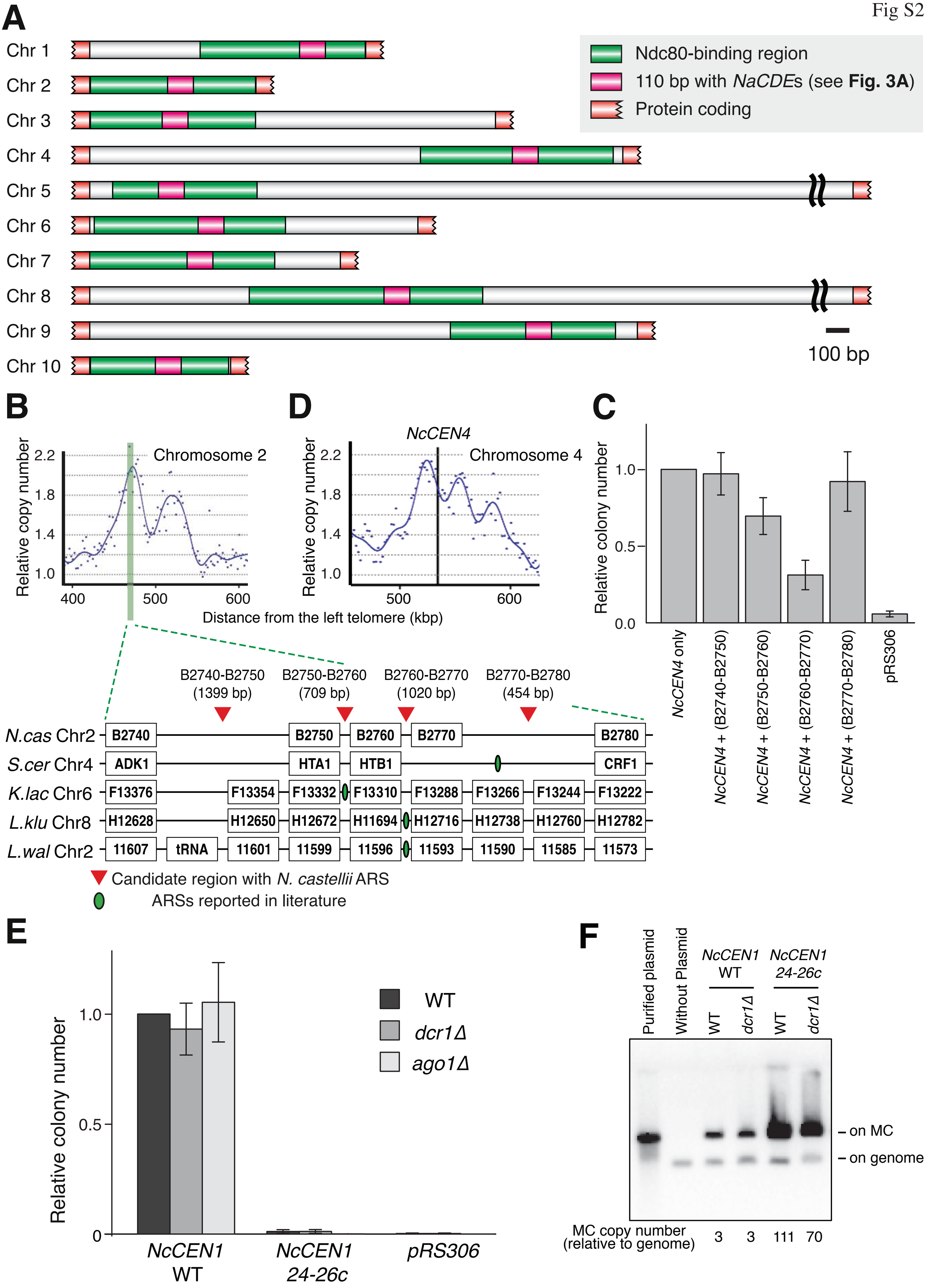
A
Chr 1
Chr 2
Chr 3
Chr 4
Chr 5
Chr 6
Chr 7
Chr 8
Chr 9
Chr 10
Ndc80-binding region
110 bp with NaCDEs (see Fig. 3A)
Protein coding
100 bp
B
Relative copy number
Chromosome 2
2.2
1.8
1.4
1.0
400
500
600
D
NcCEN4
Chromosome 4
Distance from the left telomere (kbp)
C
Relative colony number
1.0
0.5
0.0
NcCEN4 only
NcCEN4 + (B2740-B2750)
NcCEN4 + (B2750-B2760)
NcCEN4 + (B2760-B2770)
NcCEN4 + (B2770-B2780)
pRS306
B2740-B2750 (1399 bp)
B2750-B2760 (709 bp)
B2760-B2770 (1020 bp)
B2770-B2780 (454 bp)
N.cas Chr2 B2740 B2750 B2760 B2770 B2780
S.cer Chr4 ADK1 HTA1 HTB1 CRF1
K.lac Chr6 F13376 F13354 F13332 F13310 F13288 F13266 F13244 F13222
L.klu Chr8 H12628 H12650 H12672 H11694 H12716 H12738 H12760 H12782
L.wal Chr2 11607 tRNA 11601 11599 11596 11593 11590 11585 11573
Candidate region with N. castellii ARS
ARSs reported in literature
E
Relative colony number
1.0
0.5
0
WT
dcr1Δ
ago1Δ
NcCEN1 WT
NcCEN1 24-26c
pRS306
F
Purified plasmid
Without Plasmid
NcCEN1 WT
NcCEN1 24-26c
WT
dcr1Δ
WT
dcr1Δ
on MC
on genome
MC copy number (relative to genome)
3
3
111
70

Figure S2. Supplemental Figures associated with Figure 3.

(**A**) **Diagram of intergenic regions where Ndc80 is enriched.** Green bars represent chromosome regions where ChIP-seq shows enrichment of Ndc80. Orange bars show protein coding regions. Pink bars represent 110 bp DNA fragments (shown in Figure 3A) where consensus DNA sequences are identified. Scale bar, 100 bp.

(**B**–**D**) ***pRS306*-based minichromosomes may replicate efficiently in *N. castellii* cells.** We found that *pRS306* was maintained at a high-copy number in *N. castellii* cells (> 70 copies per cell; Figure 3E). Addition of *N. castellii CEN1* to *pRS306* led to a marked reduction in copy-number of the minichromosome (to 3–5 copies per cell; Figure 3E, *NcCEN1* WT) and to formation of many more yeast colonies (Figure 3B). These results suggest that *pRS306* is able to replicate in *N. castellii* cells. However, it is still unclear how efficiently this replication happens in *N. castellii* cells, and we addressed this question. If replication is not efficient, addition of an efficient replication origin may improve propagation of *pRS306* with *N. castellii CEN,* thus increasing the number of *N. castellii* colonies with this minichromosome. Replication origins have been identified in several budding yeast species [S5-8]. However, they have not yet been identified in *N. castellii*, so we used candidate DNA fragments, derived from the *N. castellii* genome, which likely carry an efficient replication origin. **B** shows replication timing profile around the histone H2 cluster (top), which is taken from a genome-wide replication timing profile of *N. castellii* (Müller and Nieduszynski, unpublished). In the replication timing profile, high and low relative copy numbers represent replication in early and late S phase, respectively [S9]. The gene order is aligned in the histone H2 cluster region of *N. castellii*, *S. cerevisiae, K. lactis, L. kluyveri* and *L. waltii* (bottom). Green ovals show positions of previously identified replication origins (ARS; autonomously replicating sequence) [S6-8]. Red arrowheads show candidate intergenic regions for the origin activity in *N. castellii.* We inserted DNA fragments of these intragenic regions into *pRS306,* together with *N. castellii CEN4* (841 bp). **C** shows results of colony formation assay. Colony formation was evaluated after introducing each minichromosome into T11421 (*ho*Δ *ura3-1*) cells. The number of colonies with each minichromosome was normalized to that with *pRS306* plus *N. castellii CEN4.* Error bar represents SEM (n=3). **D** shows replication timing profiles around *N. castellii CEN4*, which is taken from a genome-wide replication timing profile of *N. castellii* (Müller and Nieduszynski, unpublished).
We interpret the results as follows: It is likely that at least one of the four intergenic regions, shown by red arrowheads in **B**, carries an efficient replication origin, because 1) other budding yeast species have origin activity (ARS) at one of the intergenic regions of the histone H2 cluster, and this replication origin is important for the early replication of histone genes, which is thought to be required for timely histone supply during S phase [S8, S10]; and 2) there is a peak in the replication timing profile within this cluster in *N. castellii,* suggesting the presence of a replication origin. On the other hand, it is unlikely that the *N. castellii CEN4* DNA fragment carries an efficient replication origin, as there is no peak at *NcCEN4* in the replication timing profile. In the colony formation assay, the addition of *N. castellii CEN4* to *pRS306* markedly increased the number of colonies. However, none of the intergenic DNA fragments added to *pRS306* with *N. castellii CEN4* led to a further increase in colony number. Therefore, if as we predict, at least one of the intergenic DNA fragments indeed carries an efficient replication origin, it is likely that *pRS306* itself can already replicate efficiently in *N. castellii* cells. We speculate that, in *N. castellii* cells, *pRS306* may show promiscuous replication initiation from multiple sites, or it may carry an origin activity on its particular DNA sequence.

(**E, F**) **RNAi may not be required for the centromere activity in *N. castellii.*** RNA interference (RNAi) is a gene-silencing pathway triggered by double-stranded RNA [S11]. This pathway is present in some budding yeasts, including *N. castellii*, while it was lost in other budding yeasts during their evolution [S12]. Given that RNAi is important for centromere activity in fission yeast [S11], we addressed if RNAi is required for centromere activity in *N. castellii*. In **E**, *dcr1*Δ (T11420) and *ago1*Δ (T11459) strains of *N. castellii* were used for colony formation assay. Minichromosomes (MCs) with *NcCEN1* wild-type (WT), *NcCEN1* mutant 24–26c (see Figure 3C) and *pRS306* were introduced, and colony numbers were counted, as in Figure 3B, D. Colony numbers were normalized to wild-type (*WT*, *AGO1+ DCR1+*; T11421) cells with *NcCEN1* wild-type. Error bars represent SEM (n=3). Normalized colony numbers with *NcCEN1 24-26c* and *pRS306* were as follows (mean±SEM): *NcCEN1 24-26c*; 0.012±0.008 (*wild-type*), 0.012±0.009 (*dcr1*Δ), 0.001±0.001 (*ago1*Δ). *pRS306*; 0.002±0.002 (*wild-type*), 0.002±0.002 (*dcr1*Δ), 0.000±0.000 (*ago1*Δ). Thus, with wild-type *NcCEN1*, wild-type, *dcr1*Δ and *ago1*Δ strains showed similar numbers of colonies, which were reduced when the *NcCEN1* mutant was used. In **F**, we analyzed the copy number of MCs in *wild-type* and *dcr1*Δ cells, using the Southern blot. Genomic and MC DNA was digested, separated, blotted and probed (with the ampicillin resistance gene) as in Figure 3E. The ampicillin resistance gene was integrated on the genome of these cells and also carried by MCs. In both cells, MCs with wild-type *NcCEN1* showed a low copy number (3 per cell), while those with mutant *NcCEN1* showed a high copy number (70–111 per cell). Note that *ago1*Δ cells did not have the ampicillin resistance gene integrated on the genome and could not be analyze with the same probe. Collectively, these results suggest that *dcr1*Δ and *ago1*Δ cells support the activity of *NcCEN1*, as do wild-type cells. Thus, RNAi system may not be required for centromere activity in *N. castellii*. Note that Drinnenberg *et al.* originally reported that *N. castellii* has a basic RNAi system [S12], and suggested that RNAi is required for plasmid stability. Their study used *pRS316,* but we find this plasmid does not carry an active centromere for *N. castellii* (see Figure 3B, D, E); therefore we do not think their study directly addressed the RNAi requirement for centromere activity in *N. castellii.*

A

Probe L	Chromosome 10 Shortened	Chromosome 10 Unchanged
WT	10	3
21-23a	0	11
24-26c	0	7
45-47t	0	7

Probe R	Chromosome 10 Shortened	Chromosome 10 Unchanged
WT	13	0
21-23a	0	11
24-26c	0	7
45-47t	0	7

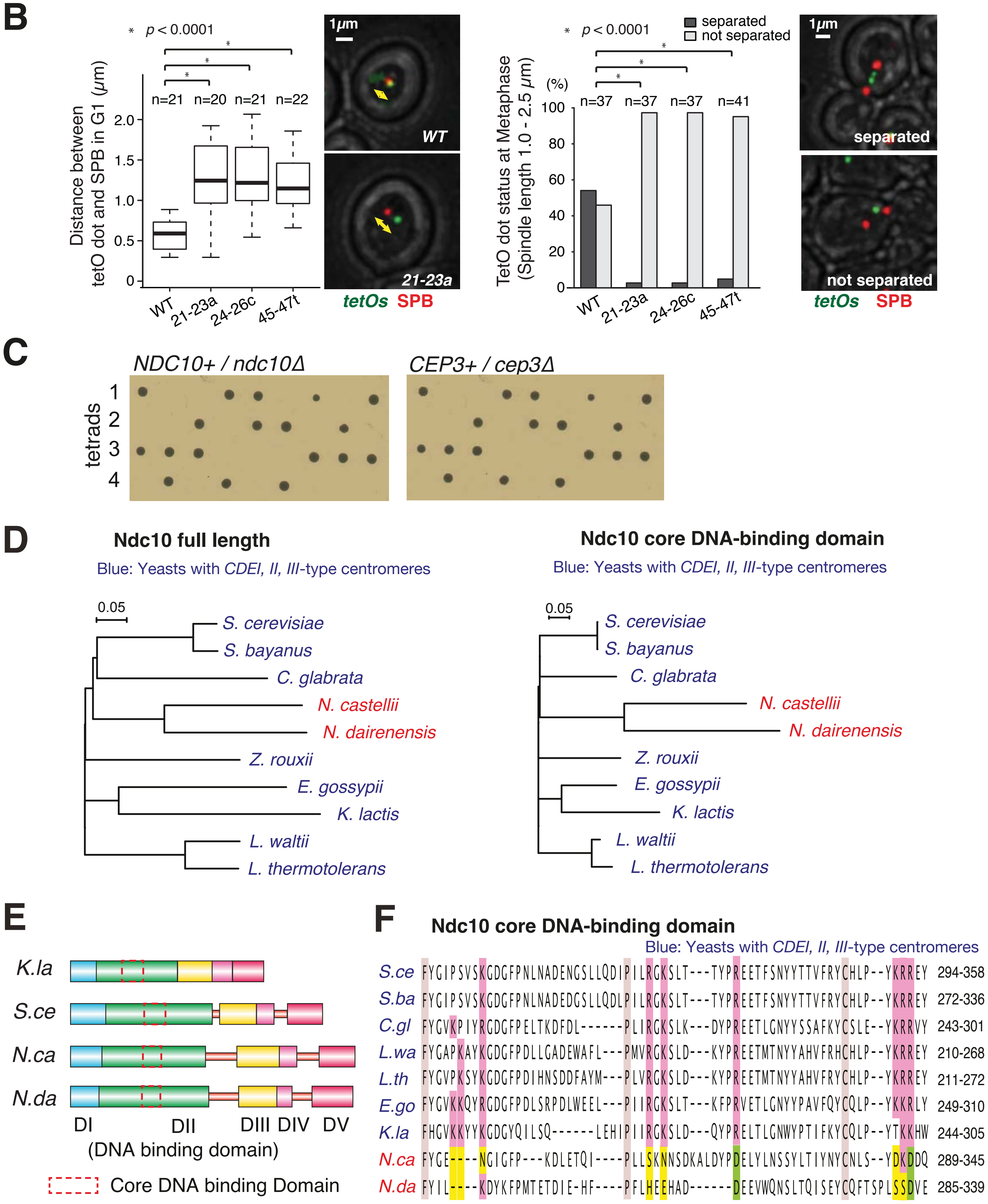

Figure S3. Supplemental Figures associated with Figure 4

(**A**) **Chromosome 10 is shortened, which is detected by ProbeL and ProbeR, after insertion of wild-type *NcCEN1* onto this chromosome.** *NcCEN1* wild-type and its mutants (21–23a, 24–26c and 45–47t: see Figure 3C) were inserted on chromosome 10 in *N. castellii* cells (Figure 4A). Karyotypes of individual clones were analyzed by pulsed field gel electrophoresis (PFGE), followed by southern blots with ProbeL and ProbeR (Figure 4A). As shown in these tables, we detect shortening (i.e. breakage) of chromosome 10 with both ProbeL and ProbeR, in most clones (10/13 and 13/13, respectively), after wild-type *NcCEN1* was inserted on an arm of chromosome 10. In these clones, the sizes of two chromosome 10 fragments, each detected by ProbeL and ProbeR, are negatively correlated; i.e. if one is large, then the other is small. The total size of the two fragments corresponds approximately to the length of the whole chromosome 10 (Figure 4A). This is consistent with chromosome 10 being broken somewhere between *NcCEN10* and the inserted *NcCEN1*. It is known that, after a dicentric chromosome is broken in *S. cerevisiae* cells, telomeres are often generated *de novo* at broken ends, allowing stable transmission of broken chromosome fragments [S13]. On the other hand, in 3/13 clones the chromosome band, hybridized by ProbeL, did not show appreciable size changes from the original chromosome 10 (e.g. the first and second clones from the left, with wild-type *NcCEN1* in Figure 4A). Given that all these clones carried smallest chromosome 10 fragments detected by ProbeR, it is possible that chromosome 10 fragments, detected by ProbeL, are shortened modestly, but their size change could not be detected by PFGE. Alternatively, a more complex chromosome rearrangement may have been involved; for example, a small region containing *NcCEN1* may have been deleted while a small chromosome fragment with *NcCEN1* is also present in the same cell (similar to T10/3 cell in Fig 3B of [S13]). After insertion of mutant *NcCEN1*, chromosome 10 did not show size changes when hybridized by ProbeL or ProbeR. In the table in Figure 4A, chromosome 10 breakage is scored when its shortening was detected by ProbeL, ProbeR or both.

(**B**) **Analyses of *tetO*s localization after *NcCEN1* wild-type and its mutants were inserted on chromosome 10.** *NcCEN1* wild-type (WT; T11632), 21–23a (see Figure 3C; T11842), 24–26c (T11843) and 45–47t (T11844), each marked with *tetOx112,* were inserted at 214 kb right of *NcCEN10* on chromosome 10 in *SPC42-4xmCherry TetR-GFP* cells, as in Figure 2B. Left: distance between the SPB and the *tetO* dot in G1 phase (unbudded cells). Right: frequency of separation and non-separation of the *tetO* dot in metaphase (cells with two SPBs 1.0–2.5 µm apart).

(**C**) **Ndc10 and Cep3 are essential genes in *N.castellii*.** T11322 (*NDC10*+ / *ndc10*Δ) and T11487 (*CEP3*+ / *cep3*Δ) diploids were sporulated, tetrads were dissected, and colonies were formed. Each column represents each group of tetrads. *ndc10*Δ and *cep3*Δ were made by replacing *NDC10* and *CEP3* coding regions with *HPH* and *NAT* marker genes, respectively. None of growing colonies contained these marker genes. The results indicate that both *NDC10* and *CEP3* are essential genes for cell viability.

(**D**) **Rapid change of the Ndc10 core DNA-binding domain during evolution of *N. castellii* and *N. dairenensis*.** Phylogenetic trees were constructed from (left) full-length Ndc10 proteins after masking highly variable regions with Gblocks (452 amino acid sites), and (right) the core DNA binding domain (red rectangles in **E** and 43 amino acid residues in **F**). Trees were constrained to have the reference species topology (Figure 1A). Branch lengths were estimated by the least-squares method using uncorrected differences. Short negative-length branches were collapsed to

zero length. Scale bars indicate 5% divergence. Comparison of the two phylogenetic trees suggests that the core DNA binding domain of Ndc10 showed more rapid change than other parts of Ndc10, during evolution of *N. castellii* and *N. dairenensis.* Note that Cse4 also binds the point centromeres of budding yeasts [S14], and it is interesting to investigate how DNA-binding domains of Cse4 changed during the evolution of budding yeast species. However, there are several different models about the organization of Cse4-containing nucleosome [S15], and DNA-binding domains or residues of Cse4 have not yet been determined with agreement. Nonetheless, some domains of Cse4 showed more rapid changes during the evolution of *N. castellii*, compared with other budding yeasts [S16].

(**E**) **Diagram of the Ndc10 domains of *K. lactis*, *S. cerevisiae*, *N. castellii* and *N. dairenensis*.** Colored boxes represent conserved domains, as reported in [S17]. Red rectangles show the Ndc10 core DNA binding domains (analyzed in **D**).

(**F**) **Most of DNA-binding residues of Ndc10 in yeasts with the *CDEI, II, III*-type centromere are not conserved in *N. castellii* and *N. dairenensis*.** Ndc10 is a component of the CBF3 complex, which binds directly the consensus DNA sequence of point centromeres ([S17-20] and references therein). Amino acid sequences of the Ndc10 core DNA-binding domain (the region shown in **E**) are aligned among several budding yeast species. Within this domain, nine basic amino acid residues (lysines and arginines) have been identified to make direct contact with DNA [S17, S20] (pink shading). Six out of the nine residues are conserved (including an exchange between lysine and arginine) among all budding yeasts carrying *CDE I, II* and *III*, whereas the three other residues are conserved in some of them. By contrast, the majority of them are not conserved in *N. castellii* or *N. dairenensis* (yellow shading); in fact, only one of the nine basic amino acid residues is conserved in *N. castellii* and *N. dairenensis* (pink shading). Notably, two of the nine lysine and arginine were replaced with aspartate (D), in both *N. castellii* and *N. dairenensis* (green shading). Other conserved amino acid residues among budding yeasts are highlighted in brown. The low level of conservation of Ndc10 DNA-binding residues in *N. castellii* and *N. dairenensis* may reflect adaptation to the new type of centromere *CDE*s evolved in these budding yeast species.

Given the above results about CBF3 components, we addressed if *N. castellii* Ndc10 or CBF3 is sufficient to recognize the *N. castellii* centromere, by explanting an *N. castellii CEN* into *S. cerevisiae* cells. We integrated *NcCEN1* into an *S. cerevisiae* chromosome arm, visualized it with *tetO*s and TetR-GFP, and expressed the following proteins from the *GALS* promoter (and also from the *S. cerevisiae NDC10* promoter for Ndc10 proteins); 1) *N. castellii* Ndc10, 2) Ndc10 chimera (*S. cerevisiae* Ndc10 with its DNA binding domain replaced with that of *N. castellii* Ndc10) and 3) all four *N. castellii* CBF3 components (i.e. Ndc10, Cep3, Ctf13 and Skp1). However, the *N. castellii CEN* was not pulled to a spindle pole in *S. cerevisiae* cells, suggesting that other factors are required to recognize *N. castellii CEN* in *S. cerevisiae* cells.

Fig S4

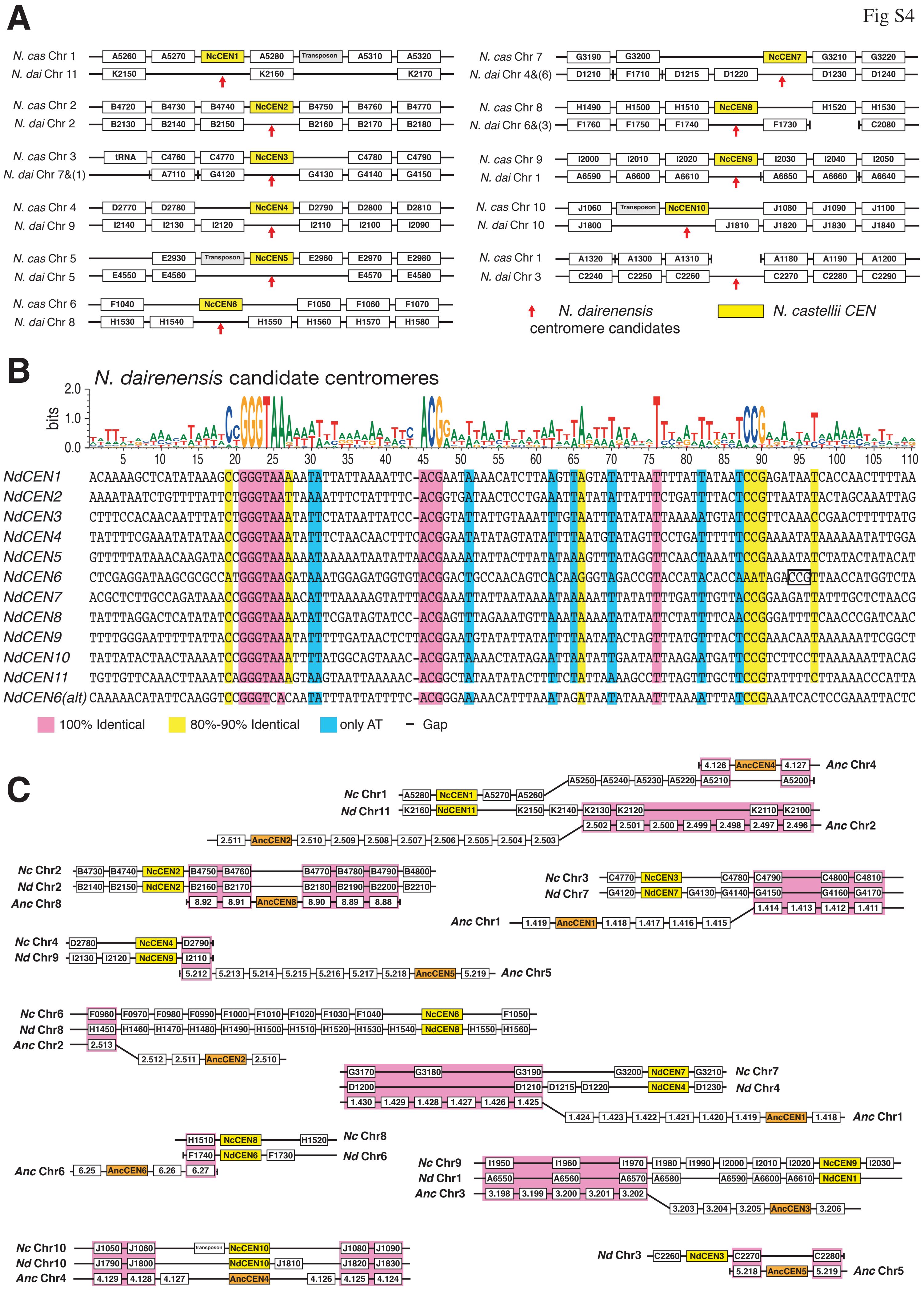
A
N. dairenensis centromere candidates
N. castellii CEN
B
N. dairenensis candidate centromeres
bits
100% Identical
80%-90% Identical
only AT
Gap
C
Anc Chr4
Anc Chr2
Anc Chr8
Anc Chr1
Anc Chr5
Anc Chr6
Anc Chr3

Figure S4. Supplemental figures associated with Figure 4

(**A**) **Gene orders in *N. castellii* and *N. dairenensis*, aligned around *N. castellii* *CEN*s**. Yellow boxes represent *N. castellii CEN*s. Red arrows indicate positions of candidate *N. dairenensis* centromeres (*NdCEN*, shown in **B**) with consensus DNA sequence (shown in Figure 4D). *N. castellii* has 10 chromosomes and *N. dairenensis* has 11. Chromosome 3 of *N. dairenensis* does not have an intergenic region corresponding to a *N. castellii CEN,* based on synteny. Nevertheless we identified a candidate centromere (*NdCEN3* in **B**) on chromosome 3, as shown in this figure.

(**B**) **Identified candidate centromere sequences (*NdCEN1–11*) on *N. dairenensis* chromosomes.** These candidate centromeres (*NdCEN*s) were identified at the *N. dairenensis* chromosome loci shown in **A** (red arrows). Logos of nucleotides at the top graphically represent their frequency at individual positions, among the *NdCEN1-11* of *N. dairenensis*. Nucleotide positions, highlighted in pink and yellow, represent those identical among 100% and 80–90% *NdCEN*s, respectively. Blue shows positions only with A and T. Consensus DNA elements, *NaCDEI, II* and *III*, are shown in Figure 4D (*N. dairenensis*). For *NdCEN6*, we identified two possible matches to the consensus in the same intergenic region: one (*NdCEN6*) that does not contain the conserved CCG at positions 88–90 but has CCG at positions 94–96 (in box), and another (*NdCEN6* [alt]) that has GGGTCA instead of the conserved GGGTAA at positions 21-26. *NdCEN6* (alt) is not included in making the sequence logo at top. *NdCEN11* is found between K2160 and K2170, but we interpret that it is at the position corresponding to *NcCEN1* (as shown in **A**) because K2160 is inverted. Note that we identified the candidate centromeres *NdCEN1-11* in *N. dairenensis*, as follows: When we used MEME to search for a consensus sequence in the *N. dairenensis* intergenic regions shown in **A** (red arrows, excluding chromosome 3), we initially found a highly conserved sequence of 251 bp that appeared to be the long terminal repeat (LTR) of a Ty-like transposable element (e.g. LTR between D1220 and D1230 on chromosome 4: European Nucleotide Archive HE580270.1, bases 291920–292170). In *N. dairenensis* there is a cluster of LTRs on each chromosome (with two clusters on chromosomes 7 and 9), and ten of these clusters map at positions syntenic with *N. castellii* centromeres, which suggested that the LTR is from a retroelement with a preference for integration at centromeres. This LTR has no counterpart in *N. castellii*. On the ‘extra’ *N. dairenensis* chromosome (chromosome 3), which contains no region orthologous to an *N. castellii* centromere, a cluster of LTRs indicated a likely centromere location. After masking LTRs and Ty fragments from these 11 candidate *N. dairenensis* intergenic regions, we identified consensus DNA elements (*CDE*s) that are very similar to *CDE*s found at *N. castellii CEN*s, as shown in this figure and Figure 4D.
Comparison of *CDE*s between *N. castellii* and *N. dairenensis* not only indicated *NaCDEI* and *II* as conserved *CDE*s, but also highlighted a third conserved *CDE* (CC at positions 88–89), which is named *NaCDEIII* (Figure 4D). ‘CCG’ in *NaCDEIII* is conserved in most candidate centromeres (10 or 11 [depending on whether including *NdCEN6*] out of 11) of *N. dairenensis* (Figure S4B). However, ‘CCG’ in *NaCDEIII* is conserved only 5 out of 10 *N. castellii* centromeres, while ‘CC’ in *NaCDEIII* is conserved 8 out of 10 (Figure 3A). Nonetheless, ‘CCG’ or ‘CC’ may still be important for *NcCEN* function when present. To test this, we mutated ‘CC’ (replaced by ‘TT’) in *NaCDEIII* of *NcCEN1*. However, this mutation reduced the colony number only modestly (64.5 ± 0.9 % of wild-type *NcCEN1*) in the assay shown in Figure 3B and D. Thus, although the ‘CC’ in *NaCDEIII* is evolutionarily conserved, it is not essential for *N. castellii* centromere function.

(**C**) **Most *N. castellii* centromeres and *N. dairenensis* candidate centromeres show direct or indirect synteny relationship to the vicinity of ancestral centromeres.** *NcCEN*s and *NdCEN*s are shown in yellow boxes, while *AncCEN*s are shown in orange boxes. Pink shading indicates that *Anc* genes and *Nc*/*Nd* genes (*Anc*: ancestor, *Nc*: *N. castellii*, *Nd*: *N. dairenensis*) in alignment are orthologous (aligned with the same gene orientation). *Nc* and *Nd* genes in alignment are also orthologous. Vertical tick bars show rearrangement between the *Anc* genome and *Nc*/*Nd* genome. Oblique lines connecting genes (boxes) show rearrangement between two genomes in comparison.

Detailed comparison to the gene order in the pre-WGD ancestor inferred by Gordon et al [S2] shows that most of *N. castellii* centromeres and *N. dairenensis* candidate centromeres show direct or indirect synteny relationship to the vicinity of ancestral centromeres as shown in this figure: *NcCEN10* and *NdCEN10* are precisely at an ancestral centromere location. *NcCEN2*, *NdCEN2* and *NdCEN3* are near, but not exactly at, an ancestral centromere position. Seven *NcCEN*s (*NcCEN1, 3, 4, 6, 7, 8, 9*) and seven *NdCEN*s (*NdCEN1, 4, 6, 7, 8, 9, 11*) have an indirect synteny relationship to the vicinity of ancestral centromeres; i.e. at least one gene, locating within 10 genes from the *NcCEN* or *NdCEN*, has an ancestral ortholog (pink shading) within 10 genes from an *AncCEN*. Only one (*NcCEN5*, not shown) is at a location that is completely unrelated to ancestral centromere locations. This result suggests that most of the ancestors of *NcCEN*s and *NdCEN*s may have been near *AncCEN* sites at some time in the past, but subsequent rearrangements in the *N. castellii* and *N. dairenensis* genome have now moved them further apart. By computer simulation, we estimate that the association between *NcCEN*s and the neighbourhoods of ancestral centromeres is statistically significant ($P < 10^{-7}$, see Experimental procedures).

If the ancestors of *NcCEN*s and *NdCEN*s were near *AncCEN* sites at some time in the past, what is the reason for it? One possible model explaining this is as follows:

The genome of a *Naumovozyma* species, prior to the divergence between *N. castellii* and *N. dairenensis*, had standard point centromeres but somehow acquired a new sequence (*NaCDEI, II, III*). At some point during evolution (before the new centromere superseded the standard point centromere), both standard point centromeres and new centromeres were functional. Because dicentric chromosomes tend to break after being caught by microtubules from the opposite spindle poles [S21], evolutionary pressure may have favoured cells with rearrangements in which the new sequence was located close to an old centromere so that two centromeres were unlikely to be caught by the opposite spindle poles. In fact, it is known that a dicentric chromosome with a shorter interval between two centromeres is more stably maintained in budding yeast [S22].

N. castellii genome
Average AT content
63.24 %

CEN Block	1-19	27-44	53-87	90-110
NcCEN1	79%	83%	86%	90%
NcCEN2	89%	94%	83%	90%
NcCEN3	89%	78%	89%	86%
NcCEN4	84%	83%	83%	86%
NcCEN5	68%	89%	83%	71%
NcCEN6	63%	94%	86%	67%
NcCEN7	68%	89%	91%	76%
NcCEN8	84%	83%	80%	76%
NcCEN9	79%	89%	83%	81%
NcCEN10	68%	83%	97%	81%

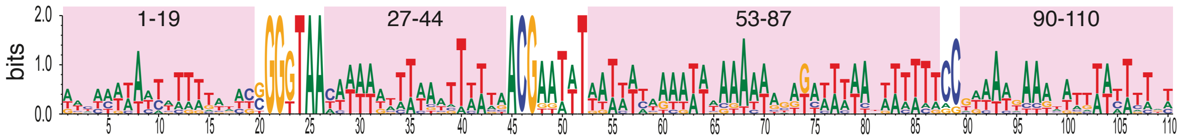

Table S1. AT contents at *N. castellii CEN*s.

Sequences of *N. castellii CEN* sequences (110 bp; see Figure 3A) were divided by three consensus sequences (*NcCDEI* [position 20-26], *NcCDEII* [position 45-52] and *CC-rich* [position 88-89]) and the percentage of AT content was analyzed in four blocks (Block 1-19, 27-44, 53-87 and 90-110). DNA sequences within these blocks are not conserved among chromosomes, but percentage of AT is higher in all blocks than the average percentage of AT on the genome (63.24% [S3]). This result is reminiscent of *CDEII* in other budding yeasts, which shows a high AT content (>79% [S19, S23, S24]).

Table S2: Yeast strains used in this study

Strain	Genotype	Parental strain		Plasmid used for construction	Through tetrad dissection	Reference Figures
**Y235**	*MATa/MATα ura3-1/ura3-1*	*N. castellii*	CBS4310			[S25] [S30]
**Y320**	*MATα ura3-1 ho*	*N. castellii*	CBS4310			[S25] [S30]
**T9377**	*MATα CSE4-6xHA::kanMX4 ura3-1 ho*	*N. castellii*	Y320	pT1729		Fig S1B, S1C
**T9328**	*MATα NDC80-6×HA::ScURA3 ho*	*N. castellii*	Y320	pT1710		Fig 1D, S1A
**T9326**	*MATα NDC10-6×HA::ScURA3 ho*	*N. castellii*	Y320	pT1709		Fig 1D, S1A
**T11322**	*MATa/MATα NDC10/ndc10Δ::hphNT1 ura3-1/ura3-1*	*N. castellii*	Y235	pT2203		Fig S3C
**T11420**	*MATα dcr1Δ::hphNT1 hoΔ::natNT2::AmpR ura3-1*	*N. castellii*	Y235	pT2205 pT2331	YES (see diagram below)	Fig S2E, S2F
**T11421**	*MATα hoΔ::natNT2::AmpR ura3-1*	*N. castellii*	Y235	pT2205 pT2331	YES (see diagram below)	Fig 3, S2C, S2E, S2F
**T11424**	*MATa/MATα NDC10-3×FLAG::kanMX4/NDC10-3×FLAG::kanMX4 ura3-1/ura3-1*	*N. castellii*	Y235	pT2330	YES (see diagram below)	diagram below
**T11450**	*MATa/MATα CEP3-3×FLAG::kanMX4/CEP3-3×FLAG::kanMX4 ura3-1/ura3-1*	*N. castellii*	Y235	pT2341	YES (see diagram below)	Fig S1B, S1C
**T11459**	*MATa/MATα ago1Δ/ago1Δ ura3-1/ura3-1*	*N. castellii*	Y235	pT2204	YES (see diagram below)	Fig S2E
**T11466**	*MATα intergenic region(J1080-J1090 next to CEN10)::tetO×112::natNT2 ura3::TetR-GFP::NcURA3 SPC42-4×mCherry::kanMX4 ho*	*N. castellii*	Y320	pT2198 pT2379 pT2367		Fig 2
**T11467**	*MATα intergenic region(J2080-J2090)::tetO×112::natNT2 ura3::TetR-GFP::NcUAR3 SPC42-4×mCherry::kanMX4 ho*	*N. castellii*	Y320	pT2198 pT2379 pT2369		Fig 2
**T11487**	*MATa/MATα CEP3/cep3Δ::natNT2 ura3-1/ura3-1*	*N. castellii*	Y235	pT2386		Fig S3C
**T11500**	*MATα intergenic region(I0780-I0790)::tetO×112::natNT2 ura3::TetR-GFP::NcURA3 SPC42-4×mCherry::kanMX4 ho*	*N. castellii*	Y320	pT2198 pT2379 pT2443		Fig 2
**T11501**	*MATα intergenic region(I2020-I2030 at CEN9)::tetO×112::natNT2 ura3::TetR-GFP::NcURA3 SPC42-4×mCherry::kanMX4 ho*	*N. castellii*	Y320	pT2198 pT2379 pT2442		Fig 2
**T11584**	*MATa/MATα CEP3/CEP3-3×GFP::hphNT1 SPC42/SPC42-4×mCherry::kanMX4 ura3-1/ura3-1*	*N. castellii*	Y235	pT2379 pT2482		Fig 1C
**T11586**	*MATa/MATα NDC80/NDC80-3×GFP::hphNT1 SPC42/SPC42-4×mCherry::kanMX4 ura3-1/ura3-1*	*N. castellii*	Y235	pT2379 pT2484		Fig 1C
**T11587**	*MATa/MATα NDC10/NDC10-3×GFP::hphNT1 SPC42/SPC42-4×mCherry::kanMX4 ura3-1/ura3-1*	*N. castellii*	Y235	pT2379 pT2485		Fig 1C
**T11605**	*MATα hoΔ::NcURA3*	*N. castellii*	Y235	pT2509	YES (see diagram below)	Fig 4A, S3A
**T11632**	*MATa/MATα intergenic region(J2080-J2090):CEN1 WT (1173bp)::tetO×112::natNT2[one allele] intergenic region(I0780-I0790)::TetR-GFP::hphNT1[one allele] SPC42/SPC42-4×mCherry::kanMX4 ura3-1/ura3-1*	*N. castellii*	Y235	pT2459 pT2379 pT2539		Fig 4B, S3B
**T11842**	*MATa/MATα intergenic region(J2080-J2090):CEN1 21-23a (1173bp)::tetO×112::natNT2[one allele] intergenic region(I0780-I0790)::TetR-GFP::hphNT1[one allele] SPC42/SPC42-4×mCherry::kanMX4 ura3-1/ura3-1*	*N. castellii*	Y235	pT2459 pT2379 pT2603		Fig 4B, S3B
**T11843**	*MATa/MATα intergenic region(J2080-J2090):CEN1 24-26c (1173bp)::tetO×112::natNT2[one allele] intergenic region(I0780-I0790)::TetR-GFP::hphNT1[one allele] SPC42/SPC42-4×mCherry::kanMX4 ura3-1/ura3-1*	*N. castellii*	Y235	pT2459 pT2379 pT2604		Fig 4B, S3B
**T11844**	*MATa/MATα intergenic region(J2080-J2090):CEN1 45-47t (1173bp)::tetO×112::natNT2[one allele] intergenic region(I0780-I0790)::TetR-GFP::hphNT1[one allele] SPC42/SPC42-4×mCherry::kanMX4 ura3-1/ura3-1*	*N. castellii*	Y235	pT2459 pT2379 pT2606		Fig 4B, S3B
**T11845**	*MATa/MATα intergenic region(J2080-J2090):CEN1 WT (1173bp)::natNT2[one allele] NDC10-3×FLAG::kanMX4/NDC10-3×FLAG::kanMX4 ura3-1/ura3-1*	*N. castellii*	T11424	pT2503		Fig 4C
**T11846**	*MATa/MATα intergenic region(J2080-J2090):CEN1 24-26c (1173bp)::natNT2[one allele] NDC10-3×FLAG::kanMX4/NDC10-3×FLAG::kanMX4 ura3-1/ura3-1*	*N. castellii*	T11424	pT2599		Fig 4C
**T11847**	*MATa/MATα intergenic region(J2080-J2090):CEN1 WT (1173bp)::natNT2[one allele] CEP3-3×FLAG::kanMX4/CEP3-3×FLAG::kanMX4 ura3-1/ura3-1*	*N. castellii*	T11450	pT2503		Fig 4C
**T11848**	*MATa/MATα intergenic region(J2080-J2090):CEN1 24-26c (1173bp)::natNT2[one allele] CEP3-3×FLAG::kanMX4/CEP3-3×FLAG::kanMX4 ura3-1/ura3-1*	*N. castellii*	T11450	pT2599		Fig 4C
**T11520**	*MATa NDC10-yEGFP::hphNT1 SPC110-mCherry::kanMX6 ho*	*S. cerevisiae*	W303			Fig 1C
**T11521**	*MATa NDC80-yEGFP::hphNT1 SPC110-mCherry::kanMX6 ho*	*S. cerevisiae*	W303			Fig 1C
**T11522**	*MATa CEP3-yEGFP::hphNT1 SPC110-mCherry::kanMX6 ho*	*S. cerevisiae*	W303			Fig 1C

Table S2 continued (diagram)

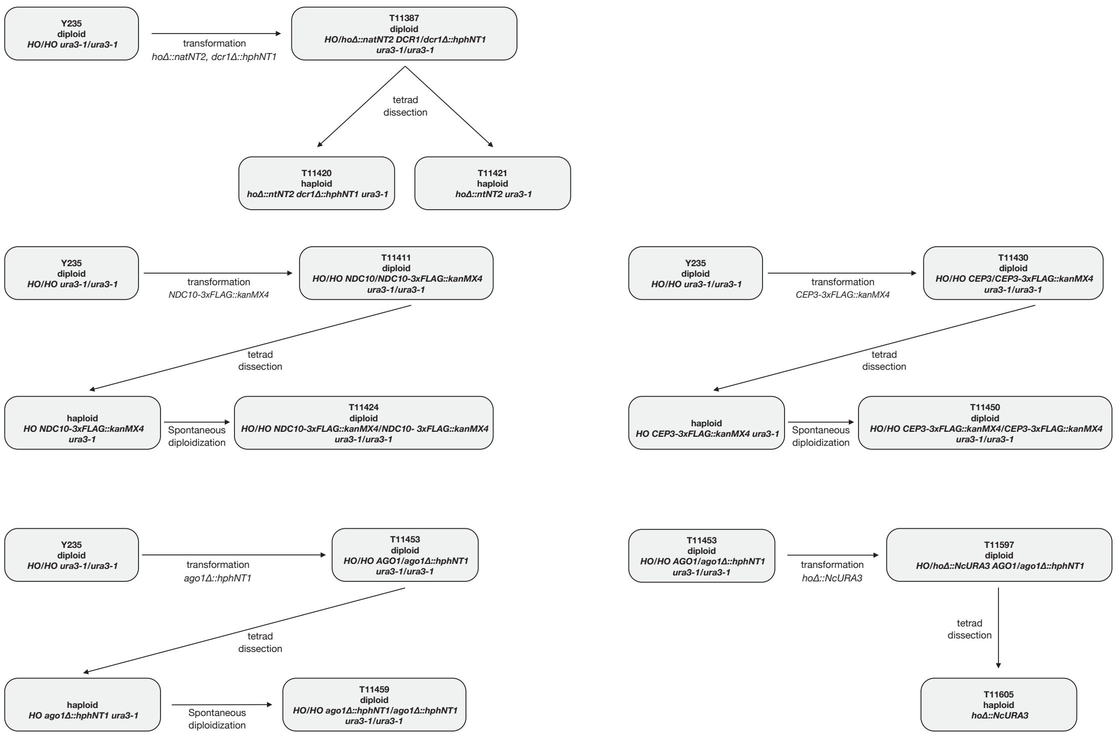

Table S3: Plasmids used for strain construction

Plasmid	Description	Integration method (see examples below)	Restriction enzymes to cut for integration
**pT1709**	*NDC10 C-terminus-6×HA::ScURA3*	ends-in	*SwaI*
**pT1710**	*NDC80 C-terminus-6×HA::ScURA3*	ends-in	*SpeI*
**pT1729**	*CSE4 C-terminus-6xHA::kanMX4::CSE4 3' UTR*	ends-out (replacement)	(PCR amplification)
**pT2198**	*NcURA3::TetR-GFP::NcURA3*	ends-in	*NheI*
**pT2203**	*NDC10 5' UTR::hphNT1:: NDC10 3' UTR (ndc10Δ)*	ends-out (replacement)	*BstBI/BamHI*
**pT2204**	*AGO1 5' UTR::hphNT1::AGO1 3' UTR (ago1Δ)*	ends-out (replacement)	*SalI/EcoRV*
**pT2205**	*DCR1 5' UTR::hphNT1:: DCR1 3' UTR (dcr1Δ)*	ends-out (replacement)	*MfeI/BglII*
**pT2330**	*NDC10 C-terminus-3×FLAG::kanMX4::NDC10 3' UTR*	ends-out (insertion)	*EcoRV/PvuII*
**pT2331**	*HO 5' UTR::natNT2::AmpR::HO 3' UTR (hoΔ)*	ends-out (replacement)	*Eco47III*
**pT2341**	*CEP3 C-terminus-3×FLAG::kanMX4::CEP3 3' UTR*	ends-out (insertion)	*PvuII/EcoRV*
**pT2367**	*intergenic region(next to CEN10)::tetO×112::natNT2*	ends-out (insertion)	*Eco47III*
**pT2369**	*intergenic region(J2080-J2090)::tetO×112::natNT2*	ends-out (insertion)	*Eco47III*
**pT2379**	*SPC42 C-terminus-4xmCherry::kanMX4::SPC42 3' UTR*	ends-out (insertion)	*SpeI/HpaI*
**pT2386**	*CEP3 5' UTR::natNT2::AmpR::CEP3 3' UTR (cep3Δ)*	ends-out (replacement)	*Eco47III*
**pT2442**	*intergenic region(at CEN9)::tetO×112::natNT2*	ends-out (insertion)	*Eco47III*
**pT2443**	*intergenic region(I0780-I0790)::tetO×112::natNT2*	ends-out (insertion)	*Eco47III*
**pT2459**	*intergenic region(I0780-I0790)::TetR-GFP::hphNT1*	ends-out (insertion)	*Eco47III*
**pT2482**	*CEP3 C-terminus-3×GFP::hphNT1::CEP3 3' UTR*	ends-out (insertion)	*EcoRV/PvuII*
**pT2484**	*NDC80 C-terminus-3×GFP::hphNT1::ND80 3' UTR*	ends-out (insertion)	*PvuII*
**pT2485**	*NDC10 C-terminus-3×GFP::hphNT1::NDC10 3' UTR*	ends-out (insertion)	*EcoRV/PvuII*
**pT2503**	*intergenic region(J2080-J2090)::CEN1 1173bp WT::natNT2*	ends-out (insertion)	*Eco47III*
**pT2509**	*HO 5' UTR::NcURA3::AmpR::HO 3' UTR (hoΔ)*	ends-out (replacement)	*Eco47III*
**pT2539**	*intergenic region(J2080-J2090)::CEN1 1173bp WT::tetO×112::natNT2*	ends-out (insertion)	*Eco47III*
**pT2598**	*intergenic region(J2080-J2090)::CEN1 1173bp (21-23a)::natNT2*	ends-out (insertion)	*Eco47III*
**pT2599**	*intergenic region(J2080-J2090)::CEN1 1173bp (24-26c)::natNT2*	ends-out (insertion)	*Eco47III*
**pT2601**	*intergenic region(J2080-J2090)::CEN1 1173bp (45-47t)::natNT2*	ends-out (insertion)	*Eco47III*
**pT2603**	*intergenic region(J2080-J2090)::CEN1 1173bp (21-23a)::tetO×112::natNT2*	ends-out (insertion)	*Eco47III*
**pT2604**	*intergenic region(J2080-J2090)::CEN1 1173bp (24-26c)::tetO×112::natNT2*	ends-out (insertion)	*Eco47III*
**pT2606**	*intergenic region(J2080-J2090)::CEN1 1173bp (45-47t)::tetO×112::natNT2*	ends-out (insertion)	*Eco47III*

pT2503, pT2598, pT2599 and pT2601 were used in Figure 4A

Ends-out (Replacement) [Example]

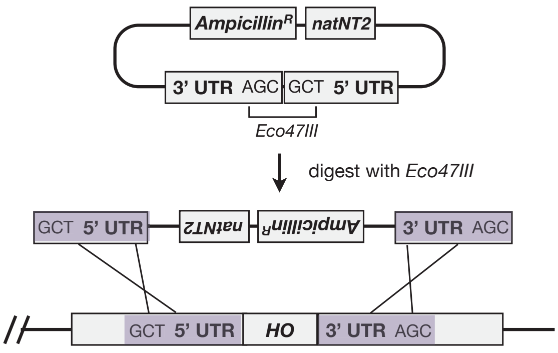

Ends-out (Insertion) [Example]

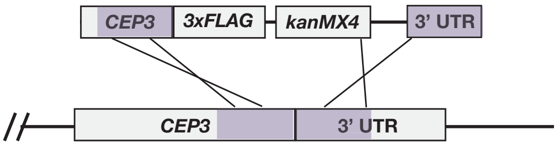

Ends-in [Example]

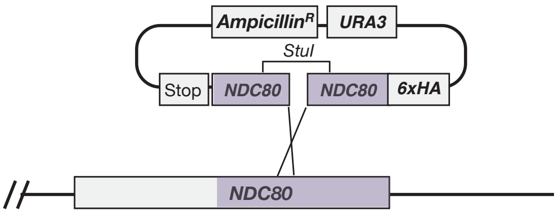

Table S4: Plasmids used for colony formation assays

Plasmid	Description	Reference Figures
**pRS316**	*ScURA3, ScCEN6 & ScARSH4*	[S41]
**pRS306**	*ScURA3*	[S41]
**pT2279**	*NcCEN1 1173 bp on pRS306*	Fig 3B, 3D, 3E, S2E, S2F
**pT2315**	*NcCEN1 110 bp on pRS306*	Fig 3B
**pT2314**	*NcCEN1 110R on pRS306*	Fig 3B
**pT2312**	*NcCEN1 110L on pRS306*	Fig 3B
**pT2302**	*NcCEN1 70 bp on pRS306*	Fig 3B
**pT2281**	*NcCEN10 589 bp on pRS306*	Fig 3B
**pT2289**	*NcCEN10 252 bp on pRS306*	Fig 3B
**pT2325**	*NcCEN10 110 bp on pRS306*	Fig 3B
**pT2304**	*NcCEN10 70 bp on pRS306*	Fig 3B
**pT2290**	*NcCEN10 off center on pRS306*	Fig 3B
**pT2670**	*NcCEN1 1173 bp 32-34c on pRS306*	Fig 3D, 3E
**pT2671**	*NcCEN1 1173 bp 37-39g on pRS306*	Fig 3D, 3E
**pT2519**	*NcCEN1 1173 bp 21-23a on pRS306*	Fig 3D, 3E
**pT2520**	*NcCEN1 1173 bp 24-26c on pRS306*	Fig 3D, 3E, S2E, S2F
**pT2524**	*NcCEN1 1173 bp 45-47t on pRS306*	Fig 3D, 3E
**pT2692**	*intergenic region 1064 bp (A5960-5970) on pRS306*	Fig 3D, 3E
**pT2240**	*NcCEN4 842 bp on pRS306*	Fig S2C
**pT2356**	*NcCEN4 842 bp, intergenic region 1399 bp (B2740-B2750) on pRS306*	Fig S2C
**pT2247**	*NcCEN4 842 bp, intergenic region 709 bp (B2750-B2760) on pRS306*	Fig S2C
**pT2248**	*NcCEN4 842 bp, intergenic region 1020 bp (B2760-B2770) on pRS306*	Fig S2C
**pT2249**	*NcCEN4 842 bp, intergenic region 454 bp (B2770-B2780) on pRS306*	Fig S2C

Sc: *Saccharomyces cerevisiae*
Nc: *Naumovozyma castellii*

Supplemental Experimental Procedures

Yeast strain construction

N. castellii strains were constructed, based on Y235 (*MATa/MATα ura3-1/ura3-1 HO/HO*) and Y320 (*MATα ura3-1 ho*); all of them derive from CBS4310 [S25]. Table S2 shows the genotypes of *N. castellii* and *S. cerevisiae* strains, used in this study. The plasmids, used for construction of *N. castellii* strains, are listed in Table S3. To generate homozygous diploid strains (T11424, T11450 and T11459), relevant constructs (*NDC10-3xFLAG, CEP3-3xFLAG and ago1*Δ*,* respectively) were integrated into Y235, which was followed by sporulation, tetrad dissection and spontaneous diploidization. *S. cerevisiae* strains, used in Figure 1C, were constructed by a one-step PCR method for gene tagging, using yEGFP-HphNT1 (pYM25 [S26]) and mCherry-KanMX6 [S27] cassettes. In both *N. castellii* and *S. cerevisiae* strains, relevant genes were tagged at their C-terminus at their original loci, and the tagged genes were expressed from their original promoters, unless otherwise stated. *N. castellii CSE4* was tagged with 6x *HA* at the C-terminus and with 3x *HA* at an internal site (between residues 107 and 108, which corresponds to residues 81–82 of *S. cerevisiae* Cse4 [S28]); both versions gave similar results in ChIP-seq.

Plasmid construction

The plasmids, used for construction of *N. castellii* strains, are listed in Table S3. Relevant 400–600 bp DNA fragments, derived from the *N. castellii* genome, were cloned into plasmids, which were integrated at target sites on the genome, using ends-in or ends-out methods [S29], as described in Table S3.

Cell growth and transformation

Yeast cells were cultured at 25ºC in YP medium containing 2% glucose (YPD). Transformations were performed as described previously [S29, S30] with some modifications: either 2 µg of circular plasmid DNA, or 2–5 µg of linear DNA was added to a mixture of 12.5 µl of single-stranded DNA (2 mg/ml salmon sperm DNA, Sigma–Aldrich D1626), 120 µl of 50% PEG 3350 (Sigma-Aldrich), 18 µl of 1 M LiAc, and mixed with ~1.5×10^8 cells. After incubation at 30ºC for 30 min, the mixtures were heat-shocked at 42ºC for 20 min and then plated on selective media. Where antibiotics were subsequently used for selection, cells were incubated for 6 h at 25ºC in YPD without antibiotics before being plated with antibiotics.

ChIP-seq and ChIP-qPCR

The procedures were based on previously described methods [S31] with some modifications. Yeast cells were cultured in 200 ml YPD medium. For crosslinking, cells were incubated with 1% formaldehyde at 25ºC for 20 min and then shifted to 4ºC overnight. Cells were harvested and washed with 100 ml sterile water and then washed with 50 ml TBS buffer (20 mM TrisCl pH 7.4, 150 mM NaCl). Cell pellets were suspended in 600 µl of lysis buffer (50 mM HEPES-KOH pH 7.5, 140 mM NaCl, 1 mM EDTA pH 8.0, 1% TritonX-100, 0.1% sodium deoxycholate and 2x protease inhibitor cocktail, cOmplete-Mini [Roche]) and frozen in liquid nitrogen. Frozen cells were then physically disrupted with a mortar and a pestle. Cell powders were resuspended in 600 µl of lysis buffer, and sonicated with Bioruptor (Diagenode; 30 sec x 30 cycles with 30 sec intervals at high intensity), resulting in an average DNA fragment size of 200 bp. The lysates were clarified by centrifugation at 13,000 g for 15 min. Aliquots of 40 µl of the lysate were used to prepare whole-cell extract (WCE) DNA. The remaining lysate was used for immunoprecipitation, Anti-FLAG antibody (M2; Sigma-Aldrich) or Anti-HA antibody (HA.11; Covance) were pre-incubated with 30 µl magnetic beads (Dynabeads Protein A for anti-HA antibody and Protein G for anti-FLAG antibody), which were then added to cell lysates and

incubated at 4ºC overnight. After incubation, the magnetic beads were washed twice with 1 ml of lysis buffer, twice with 1 ml of lysis buffer with 360 mM NaOH, twice with 1 ml of wash buffer (10 mM TrisCl pH8.0, 250 mM LiCl, 0.5% NP-40, 0.5% sodium deoxycholate, 1 mM EDTA) and finally once with 1 ml TE. Then, 40 µl of elution buffer (1% SDS in TE) was added, followed by incubation at 65ºC for 15 min. To reverse the crosslinks, the immunoprecipitated DNA and input DNA in the WCE were incubated at 65ºC overnight, and then treated with 10 µg of RNase A (Sigma–Aldrich) and 100 µg of Proteinase K (Roche) at 37ºC for 2 h. DNA was recovered using a MinElute PCR purification kit (QIAGEN). For ChIP-seq, immunoprecipitated DNA and input DNA in WCE were sequenced using Illumina GAIIx, as previously described [S32]. Sequence tags (36 bases) were mapped on the annotated *N. castellii* genome (CBS 4309) [S3] using ELAND software (Illumina), and counted using BEDTools [S33] at 100 bp no-overlapping windows. The ChIP/input ratio was obtained at each window and plotted using R software (ver 3.0.1) [S34]. ChIP-seq data are absent at some chromosome regions where DNA sequence is repetitive. For quantitative PCR (qPCR), immunoprecipitated DNA and input DNA in WCE were analyzed by Rotor-Gene 6000 (QIAGEN) and SYBR Green PCR kit (QIAGEN).

Live cell imaging
In all microscopy experiments, live yeast cells were observed without fixation. The procedures for time-lapse fluorescence microscopy were described previously [S35]. Cells were grown in YPD medium and suspended in synthetic medium containing 2% glucose for imaging. Time-lapse images were collected at 25ºC. For image acquisition, we used a DeltaVision Elite microscope (Applied Precision), UPlanSApo 100x objective lens (Olympus; NA 1.40), CoolSnap HQ2 CCD camera (Photometrix), and SoftWoRx software (Applied Precision). Images were analyzed with Volocity software (PerkinElmer).

Computer program and statistical analyses
Multiple sequences of amino acid residues were aligned using JalView (Ver 2.8) [S36] and MafftWS (version 6.8.57) [S37]. The Ndc10 full-length alignment was filtered with Gblocks [S38] to remove unreliably aligned regions. Statistical analyses were carried out using R software (ver 3.0.1) [S34] by choosing the unpaired *t*-test (Figures 2C, D, F; 4B; S3B left) or Fisher's exact test (Figures 2E, S3B right). Gene order along chromosomes was analyzed using YGOB (Yeast Gene Order Browser) [S1]. Centromere motifs in *N. castellii* and *N. dairenensis* were identified using MEME (http://meme.nbcr.net/meme/) [S39]. *N. dairenensis* LTR elements and Ty fragments were identified manually and masked before MEME analysis. To create logos of nucleotides (Figures 1B, 4D and S4B), we used the Weblogo3 program (http://weblogo.threeplusone.com/) [S40]. Box plots (Figures 2C, D, F; 4B: S3B left) were made by the 'boxplot' in R software. Box indicates the value from the 1st to 3rd quartile (interquartile range: IQR) and a thick line in the box shows a median. The upper whisker and lower whisker show the maximum and minimum values, respectively, which do not exceed 3/2 IQR beyond the box. Outliers, which exceed the range between whiskers, are shown individually in open circles. To estimate the significance of the association between *NcCEN*s and the neighbourhoods of ancestral centromeres (Figure S4C), we carried out 10 million replicate simulations in which a random intergenic region was chosen from each *N. castellii* chromosome, and the number of these that were <10 genes away from an *N. castellii* gene whose ortholog in the ancestral genome is <10 genes from an ancestral centromere was counted. The number of associations seen in the real genome (9) was not matched or exceeded in any of the replicates. The median number of associations in the simulations was 1.

Colony formation assay and copy number analysis

The plasmids, used in colony formation assays, were constructed by cloning DNA fragments into the *pRS306* plasmid [S41] and are listed in Table S4. The plasmids (2 µg) were incubated with T11421 (*ho*Δ *ura3-1*) cells for transformation; T11420 (*dcr1*Δ, *ura3-1*) and T11459 (*ago1*Δ, *ura3-1*) cells were also used in Figure S2E. Transformed cells were plated on uracil dropout with 2% glucose. After two days, colonies were counted. For copy number analysis, a single colony was picked and re-plated on uracil dropout plate. One day later, cells were inoculated into 25 ml of uracil dropout media and cultured overnight. To isolate genomic and plasmid DNA from cells, 2 x 10^8 cells were processed with Dr GenTLE High Recovery kit (TaKaRa) following the manufacturer's instructions. Genomic and plasmid DNA was digested with *NotI*, separated on a 1% agarose gel and blotted onto nylon membrane (GE Healthcare). The probes for Southern blots were labeled with Biotin-11-dUTP using North2South Biotin Random Prime Labeling Kit (Thermo Scientific). The hybridization and detection were carried out using North2South Chemiluminescent Detection system (Thermo Scientific) following the manufacturer's protocol. Chemiluminescent signal was detected using Bio-Rad ChemDoc, and images were analyzed quantitatively using Image Lab Software (Bio-Rad).

Pulsed field gel electrophoresis (PFGE)

After overnight culture, cells in 3 ml YPD (OD600 ≈ 1.0) were collected and washed with 1 ml sterile water and then with 1 ml of 0.5 M EDTA. Subsequently cells were suspended in 20 µl of Zymolyase solution (2 mg/ml Zymolyase 100T, 0.5 mM sorbitol, 100 mM $NaPO_4$, 10 mM EDTA) and incubated for 10 min at 37ºC. Low melting point agarose (80 µl of 2%) was added and the mixture was pipetted into disposable CHEF plug molds (Bio-Rad) and solidified at 4ºC. Plugs were then rinsed twice with 0.5 mM EDTA, and incubated with 1 ml of Proteinase K solution (0.5 M EDTA, 1% w/v sarkosyl, 2 mg/ml Proteinase K [Roche]) at 50ºC overnight. Subsequently, plugs were washed twice with 50 mM EDTA, twice with 0.5 x TBE (50 mM Tris-Cl, 50 mM boric acid, 1 mM EDTA) and stored at 4ºC. Chromosomes were separated by PFGE using the CHEF-DR II System (Bio-Rad), with switch time of 6.8–158 sec and 6 V/cm voltage, for 24 hours at 16ºC. PFGE markers were run as reference. The gels were strained with SYBR® Safe (Invitrogen) and scanned with a Fujifilm FLA-5100 Image Scanner. Southern blots were as described in the previous section. Probes for the Southern blots were prepared by amplifying relevant DNA sequences using PCR, with the genome DNA as a template.

Supplemental References

S1. Byrne, K.P., and Wolfe, K.H. (2005). The Yeast Gene Order Browser: combining curated homology and syntenic context reveals gene fate in polyploid species. Genome Res. *15*, 1456-1461.

S2. Gordon, J.L., Byrne, K.P., and Wolfe, K.H. (2009). Additions, losses, and rearrangements on the evolutionary route from a reconstructed ancestor to the modern Saccharomyces cerevisiae genome. PLoS Genet. *5*, e1000485.

S3. Gordon, J.L., Armisen, D., Proux-Wera, E., OhEigeartaigh, S.S., Byrne, K.P., and Wolfe, K.H. (2011). Evolutionary erosion of yeast sex chromosomes by mating-type switching accidents. Proceedings of the National Academy of Sciences of the United States of America *108*, 20024-20029.

S4. Marie-Nelly, H., Marbouty, M., Cournac, A., Liti, G., Fischer, G., Zimmer, C., and Koszul, R. (2014). Filling annotation gaps in yeast genomes using genome-wide contact maps. Bioinformatics. *30*, 2105-2113.

S5. Nieduszynski, C.A., Knox, Y., and Donaldson, A.D. (2006). Genome-wide identification of replication origins in yeast by comparative genomics. Genes & development *20*, 1874-1879.

S6. Liachko, I., Bhaskar, A., Lee, C., Chung, S.C., Tye, B.K., and Keich, U. (2010). A comprehensive genome-wide map of autonomously replicating sequences in a naive genome. PLoS genetics *6*, e1000946.

S7. Liachko, I., Tanaka, E., Cox, K., Chung, S.C., Yang, L., Seher, A., Hallas, L., Cha, E., Kang, G., Pace, H., et al. (2011). Novel features of ARS selection in budding yeast Lachancea kluyveri. BMC Genomics *12*, 633.

S8. Di Rienzi, S.C., Lindstrom, K.C., Mann, T., Noble, W.S., Raghuraman, M.K., and Brewer, B.J. (2012). Maintaining replication origins in the face of genomic change. Genome research *22*, 1940-1952.

S9. Müller, C.A., and Nieduszynski, C.A. (2012). Conservation of replication timing reveals global and local regulation of replication origin activity. Genome research *22*, 1953-1962.

S10. Omberg, L., Meyerson, J.R., Kobayashi, K., Drury, L.S., Diffley, J.F., and Alter, O. (2009). Global effects of DNA replication and DNA replication origin activity on eukaryotic gene expression. Mol Syst Biol *5*, 312.

S11. Lejeune, E., and Allshire, R.C. (2011). Common ground: small RNA programming and chromatin modifications. Curr Opin Cell Biol. *23*, 258-265. doi: 210.1016/j.ceb.2011.1003.1005. Epub 2011 Apr 1017.

S12. Drinnenberg, I.A., Weinberg, D.E., Xie, K.T., Mower, J.P., Wolfe, K.H., Fink, G.R., and Bartel, D.P. (2009). RNAi in budding yeast. Science. *326*, 544-550.

S13. Jager, D., and Philippsen, P. (1989). Stabilization of dicentric chromosomes in Saccharomyces cerevisiae by telomere addition to broken ends or by centromere deletion. The EMBO journal *8*, 247-254.

S14. Meluh, P.B., Yang, P., Glowczewski, L., Koshland, D., and Smith, M.M. (1998). Cse4p is a component of the core centromere of Saccharomyces cerevisiae. Cell *94*, 607-613.

S15. Padeganeh, A., De Rop, V., and Maddox, P.S. (2013). Nucleosomal composition at the centromere: a numbers game. Chromosome Res. *21*, 27-36. doi: 10.1007/s10577-10012-19335-10577. Epub 12013\ Jan 10518.}.

S16. Baker, R.E., and Rogers, K. (2006). Phylogenetic analysis of fungal centromere H3 proteins. Genetics. *174*, 1481-1492. Epub 2006 Oct 1488.

S17. Cho, U.S., and Harrison, S.C. (2012). Ndc10 is a platform for inner kinetochore assembly in budding yeast. Nat Struct Mol Biol *19*, 48-55.

S18. Hyman, A.A., and Sorger, P.K. (1995). Structure and function of kinetochores in budding yeast. Annual review of cell and developmental biology *11*, 471-495.

S19. Meraldi, P., McAinsh, A.D., Rheinbay, E., and Sorger, P.K. (2006). Phylogenetic and structural analysis of centromeric DNA and kinetochore proteins. Genome biology *7*, R23.
S20. Perriches, T., and Singleton, M.R. (2012). Structure of yeast kinetochore Ndc10 DNA-binding domain reveals unexpected evolutionary relationship to tyrosine recombinases. J Biol Chem. *287*, 5173-5179.
S21. Brock, J.A., and Bloom, K. (1994). A chromosome breakage assay to monitor mitotic forces in budding yeast. J Cell Sci. *107*, 891-902.
S22. Koshland, D., Rutledge, L., Fitzgerald-Hayes, M., and Hartwell, L.H. (1987). A genetic analysis of dicentric minichromosomes in Saccharomyces cerevisiae. Cell. *48*, 801-812.
S23. Clarke, L., and Carbon, J. (1985). The structure and function of yeast centromeres. Annu Rev Genet. *19*, 29-55.
S24. Gordon, J.L., Byrne, K.P., and Wolfe, K.H. (2011). Mechanisms of chromosome number evolution in yeast. PLoS Genet. *7*, e1002190.
S25. Petersen, R.F., Langkjaer, R.B., Hvidtfeldt, J., Gartner, J., Palmen, W., Ussery, D.W., and Piskur, J. (2002). Inheritance and organisation of the mitochondrial genome differ between two Saccharomyces yeasts. J Mol Biol *318*, 627-636.
S26. Janke, C., Magiera, M.M., Rathfelder, N., Taxis, C., Reber, S., Maekawa, H., Moreno-Borchart, A., Doenges, G., Schwob, E., Schiebel, E., et al. (2004). A versatile toolbox for PCR-based tagging of yeast genes: new fluorescent proteins, more markers and promoter substitution cassettes. Yeast *21*, 947-962.
S27. Snaith, H.A., Samejima, I., and Sawin, K.E. (2005). Multistep and multimode cortical anchoring of tea1p at cell tips in fission yeast. The EMBO journal *24*, 3690-3699.
S28. Wisniewski, J., Hajj, B., Chen, J., Mizuguchi, G., Xiao, H., Wei, D., Dahan, M., and Wu, C. (2014). Imaging the fate of histone Cse4 reveals de novo replacement in S phase and subsequent stable residence at centromeres. Elife. *3:e02203.*, 10.7554/eLife.02203.
S29. Astromskas, E., and Cohn, M. (2009). Ends-in vs. ends-out targeted insertion mutagenesis in Saccharomyces castellii. Curr Genet *55*, 339-347.
S30. Astromskas, E., and Cohn, M. (2007). Tools and methods for genetic analysis of Saccharomyces castellii. Yeast. *24*, 499-509.
S31. Tanaka, T., Knapp, D., and Nasmyth, K. (1997). Loading of an Mcm protein onto DNA replication origins is regulated by Cdc6p and CDKs. Cell *90*, 649-660.
S32. Yamashita, R., Sathira, N.P., Kanai, A., Tanimoto, K., Arauchi, T., Tanaka, Y., Hashimoto, S., Sugano, S., Nakai, K., and Suzuki, Y. (2011). Genome-wide characterization of transcriptional start sites in humans by integrative transcriptome analysis. Genome research *21*, 775-789.
S33. Quinlan, A.R., and Hall, I.M. (2010). BEDTools: a flexible suite of utilities for comparing genomic features. Bioinformatics. *26*, 841-842. doi: 810.1093/bioinformatics/btq1033. Epub 2010 Jan 1028.
S34. Team, R.C. (2013). R: A language and environment for statistical computing, (Vienna, Austria: R Foundation for Statistical Computing).
S35. Tanaka, K., Kitamura, E., and Tanaka, T.U. (2010). Live-cell analysis of kinetochore-microtubule interaction in budding yeast. Methods *51*, 206-213.
S36. Waterhouse, A.M., Procter, J.B., Martin, D.M., Clamp, M., and Barton, G.J. (2009). Jalview Version 2--a multiple sequence alignment editor and analysis workbench. Bioinformatics *25*, 1189-1191.
S37. Katoh, K., and Toh, H. (2010). Parallelization of the MAFFT multiple sequence alignment program. Bioinformatics *26*, 1899-1900.

S38. Talavera, G., and Castresana, J. (2007). Improvement of phylogenies after removing divergent and ambiguously aligned blocks from protein sequence alignments. Syst Biol. *56*, 564-577.

S39. Bailey, T.L., and Elkan, C. (1994). Fitting a mixture model by expectation maximization to discover motifs in biopolymers. Proceedings / ... International Conference on Intelligent Systems for Molecular Biology ; ISMB. International Conference on Intelligent Systems for Molecular Biology *2*, 28-36.

S40. Crooks, G.E., Hon, G., Chandonia, J.M., and Brenner, S.E. (2004). WebLogo: a sequence logo generator. Genome research *14*, 1188-1190.

S41. Sikorski, R.S., and Hieter, P. (1989). A system of shuttle vectors and yeast host strains designed for efficient manipulation of DNA in Saccharomyces cerevisiae. Genetics. *122*, 19-27.
